# Partner-sourced haptic feedback rather than environmental inputs drives coordination improvement in human dyadic collaboration

**DOI:** 10.1038/s41598-025-27258-5

**Published:** 2025-11-18

**Authors:** Yiming Liu, Raz Leib, William Dudley, Ali Shafti, A. Aldo Faisal, David W. Franklin

**Affiliations:** 1 Neuromuscular Diagnostics, TUM School of Medicine and Health, Technical University of Munich, Munich, 80809 Germany; 2 Brain & Behaviour Lab, Department of Computing and Department of Bioengineering, Imperial College London, London, SW7 2AZ United Kingdom; 3 Chair in Digital Health and Data Science, University of Bayreuth, Bayreuth, 95447 Germany; 4 Munich School of Robotics and Machine Intelligence (MIRMI), Technical University of Munich, Munich, 80992 Germany; 5 Munich Data Science Institute (MDSI), Technical University of Munich, Garching, 85748 Germany

**Keywords:** Motor control, Sensorimotor processing

## Abstract

Haptic communication is a critical communication channel in physical collaboration. However, most studies focus on simplistic tasks with predetermined solutions, restricting the exploration of collaborative behaviors. In this study, we designed a complex task derived from the classic ball-beam control problem, requiring pairs of participants to collaboratively control an unstable object with internal degrees of freedom. The task’s redundant nature allowed diverse strategies and collaboration patterns to emerge. We systematically examined the impact of different sources of haptic feedback, distinguishing between forces arising from the collaborator’s actions and forces arising from the task dynamics. Participants collaborated across five haptic feedback conditions: full haptics, partner only, environment only, no haptics, or unrelated haptics. Haptic feedback significantly enhanced interpersonal coordination, primarily via partner-sourced rather than environment-sourced haptic feedback. Despite equal access to information and mechanical leverage, participants naturally assumed leader-follower roles, which were robustly quantified across multiple metrics. We showed how leadership dynamics evolved throughout the experiment and varied across different haptic conditions. This study advances the understanding of haptic communication, highlighting the value of partner haptics in developing more effective and adaptive strategies for both human-human and human-robot collaboration.

## Introduction

Robotic research has seen remarkable advancements over recent decades. Propelled by breakthroughs in hardware and control algorithms and fused with the power of artificial intelligence, robots are able to undertake a broader spectrum of activities. This sets the stage for more sophisticated human-robot collaboration. Currently, human-robot collaboration typically involves working toward a common goal in a divisible task, with each performing separate subtasks. For example, in assembly lines, robots handle physical labor while humans perform quality control inspections. This arrangement is termed co-activity rather than true collaboration^1^. Direct physical engagement, in which robots act not only as machines but as interactive partners, is less common. While task-specific control algorithms and communication interfaces facilitate physical collaboration for certain tasks, they lack the versatility for general applications.

Recognizing these limitations, researchers aim to develop more dexterous and intelligent robotic systems that can interpret human actions, adjust their behavior dynamically, and collaborate seamlessly with humans^2–4^. A crucial objective is to establish intuitive and efficient communication channels^5^. Sperber and Wilson^6^ differentiated two types of human communication: coded communication and ostensive-inferential communication. In coded communication, a communicator encodes a message into a signal that the addressee decodes, while ostensive-inferential communication relies on people’s ability to express and recognize intentions. Ostensive-inferential communication lays the foundation for sophisticated social interactions that are uniquely human, facilitating more effective sharing of information and intentions, which enhances cooperation and social bonding^7^. While most current human-robot interfaces rely on coded communications, recent research underscores the potential benefits of integrating sensorimotor communication^8,9^. In physical collaboration, humans continuously monitor multiple sensory cues to observe their partners’ actions^10,11^, predict their intentions, and respond accordingly for better coordination^12–15^. Recent studies have shown that haptic feedback is a key communication channel in physical collaboration between humans. Partners can infer each other’s movement intentions^16,17^, and adopt complementary roles^1,18,19^ via haptic signals. Our previous work^20^ found that, during physical collaboration, followers can use haptic feedback to better coordinate with the leader, whereas leaders appear less responsive to haptic feedback. In this experiment, visual feedback was always available, while haptic feedback was either present or absent. When participants have full visual feedback, but no haptic feedback, the leader-follower asymmetry intensifies, with the leader becoming more dominant and the follower more passive^20^. Similar effects on role distribution have also been shown when manipulating the visual feedback while keeping haptic feedback constant^21^.

Previous studies on haptic communication often focused on simplified tasks with clear “correct” or “optimal” solutions, such as tracking a moving cursor^16,17^, reaching for static targets^19,21^, or rotating a crank^18^. These controlled settings help reduce human variability and facilitate analysis. However, they also constrain participants to converge toward uniform strategies, leaving little space to explore diversified collaboration strategies. It is unclear if the findings from simple tasks transfer to more challenging tasks where coordination is more critical. Furthermore, current studies often simulate haptic feedback solely through couplings between partners, such as virtual springs, ignoring the multiple simultaneous feedback sources commonly encountered in everyday tasks. For example, when two people carry a table, they feel haptic feedback not only from their partner but also from the environment (the table’s weight and collisions). Yet, little research has explored how humans differentiate and integrate multi-source haptic feedback. Addressing this gap requires studying haptic communication in tasks that reflect the complexity and ambiguity of everyday scenarios.

In this study, we investigated how haptic feedback influences interpersonal coordination, focusing on whether feedback from the environment and the partner have similar or distinct effects, and how these effects relate to role distribution in collaboration. For this, we designed a complex manipulation task that required two individuals to work closely together while receiving force feedback from both their partner and the environment. Using a haptically augmented virtual reality setup, we could selectively provide or block each source of force signals, allowing us to isolate and examine the contribution of each feedback source.

**Fig. 1:**
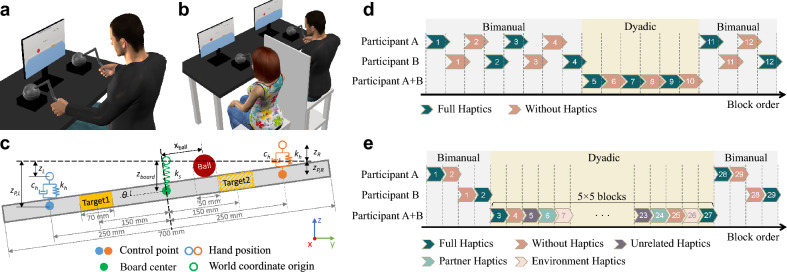
The experimental setup and protocol. (**a**) The bimanual experimental setup. (**b**) The dyadic experimental setup. (**c**) The dynamics of the virtual experiment model. (**d**) An example protocol of Experiment 1. (**e**) An example protocol of Experiment 2. In (**d**) and (**e**), the numbers inside each block mark the order of blocks experienced by each participant. The order of the haptic condition was randomized for each participant in both experiments.

## Results

Based on a task derived from the classic ball-beam problem^22^, participants (Thirty right-handed adults) used two haptic devices to control a board with a ball on it in a virtual environment. The goal was to roll the ball into a target area and stabilize it as quickly as possible (Fig. 1a-c). The target alternated between two positions on the board across trials. At the start of each trial, the ball was fixed in one target position, and participants were required to move it to the opposite target. The start and final targets were then reversed in the next trial. Participants manipulated the board by moving the two ends up and down, either bimanually, with one person controlling both sides of the board, or dyadically, with two participants each using their dominant hand to control one side of the board. In the dyadic condition, partners sat side by side, separated by a curtain to remove visual cues of the partner, with no verbal communication. The task was inherently unstable, as the ball was highly responsive to small deviations, requiring participants to react quickly and coordinate precisely. The center of the board was not fixed, so each side could move relatively independently without being constrained by the other side, resulting in redundant control and diverse collaborative strategies.

We systematically manipulated the force feedback participants received across five haptic conditions: Full (complete interaction forces), Without (no force), Partner (forces exclusively from partner’s movement), Environment (forces exclusively from environmental dynamics), and Unrelated (pre-recorded forces unrelated to the ongoing task). Experiment 1 (10 participants, 5 dyads) compared the Full and Without conditions, while Experiment 2 (20 participants, 10 dyads) extended the comparison to all five conditions. Our results show that haptic feedback enhanced coordination primarily via partner-sourced rather than environment-sourced signals. Participants naturally adopted leader-follower roles, with the role distribution evolving over time and varying across haptic conditions. These findings advance our understanding of haptic communication in complex scenarios and inform the design of more adaptive and efficient collaborative robots.

### Experiment 1

Experiment 1 examined two haptic conditions (‘Full’ and ‘Without’) across three sessions. Fig. 1d shows an example block sequence. The first and third sessions involve bimanual manipulation, with each participant completing four blocks in the first session and two in the third. The second session with six blocks examines dyadic collaboration. In both bimanual and dyadic sessions, the ‘Full’ and ‘Without’ conditions alternated between successive blocks, with the starting condition randomized for each participant (in bimanual sessions) or for each dyad (in dyadic sessions). Each block consists of 60 successful trials under one haptic condition. Across all participants, 5400 trials were completed successfully, while 89 trials failed because the ball rolled off the board. The failed trials were excluded from further analysis.

#### Completion Time

**Fig. 2:**
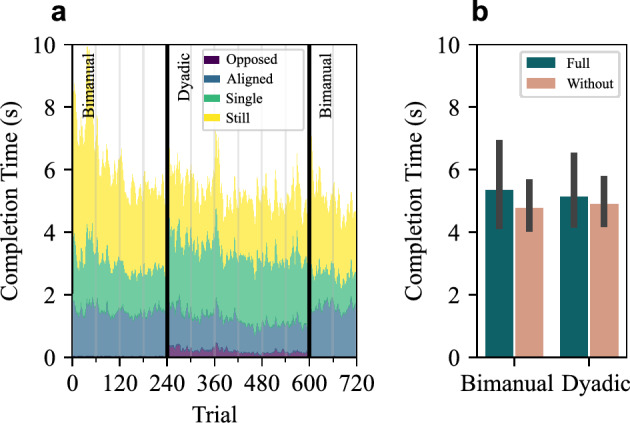
Completion time for Experiment 1. (**a**) The average completion time across all participants as a function of trial number. Each participant performed 720 trials in 12 blocks. Trials 1-240 and 601-720 were performed bimanually, whereas trials 241-600 were under dyadic conditions. Colors indicate the average time spent performing each type of interaction dynamics: aligned (two sides aligned in rotating the board), opposed (two sides opposed each other’s board rotation), single (only one side moved), and stationary (neither side moved). Detailed definitions in *Methods-Coordination*. (**b**) The completion time (mean and 95% confidence intervals) for the stable performance phase captured during the last two blocks of each of the four conditions.

Participants were instructed to complete the task as fast as possible, making completion time a straightforward measure of task performance. Completion time as a function of trial count is shown in Fig. 2a. We observed a reduction in completion times over the first two blocks (Trials 1–120), after which performance stabilized. Note that Fig. 2a averages by trial count only, without separating haptic conditions. Due to randomization, the same trial number may reflect different haptic conditions across participants. Participants reached similarly stable performance across all four conditions. Fig. 2b shows the average completion times from the last two blocks experienced under each condition, defined by pairing (Bimanual/Dyadic) and haptic feedback (Full/Without). Analysis using linear mixed models found no significant main effect of haptic condition (F(1, 13)=1.935, p=0.188), pairing condition (F(1, 13)=0.002, p=0.962), or their interaction (F(1, 13)=0.349, p=0.565). In other words, the presence of haptic feedback did not lead to improved task performance. This result is consistent with our previous experiments^20^, but contrasts previous studies that suggested haptic communication improves performance^17,23,24^. The absolute force feedback in the ‘Full’ condition was 0.33 ± 0.26 N (mean ± STD). Although these forces are low, the absence of an effect on completion time is unlikely due to the forces being below the perceptual threshold. It has been shown that detection thresholds are between 2.4-5.8 mN^25^ and force direction can be consciously identified at 0.05 N^26^.

#### Coordination

Although haptic feedback did not affect completion time, we next examined whether it influenced how participants coordinated. As the ball was highly sensitive to board tilt, the task required precise coordination between the two sides of the board. This demand was especially challenging in the dyadic condition, where partners needed to continuously adjust to each other’s movements. We assessed the coordination using two measures: interaction dynamics and delay.

Since both sides of the board could move independently, we used interaction dynamics to quantify how closely the two sides shared a common movement intent for controlling the board’s tilt. At each time point, interaction dynamics were classified into four types: aligned (both sides act toward the same rotational direction), opposed (they act in opposition), single (only one side is active), and stationary (neither side moves). For details of the calculation, see *Methods – Interaction dynamics*.

**Fig. 3:**
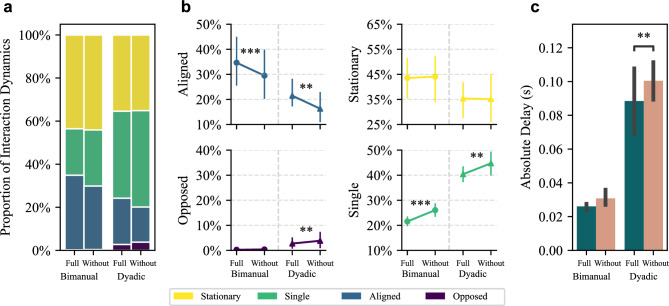
Movement classification of interaction dynamics for Experiment 1. (**a**) The proportion of each type of interaction dynamics across all trials. (**b**) The average and 95% confidence interval of the proportion of each type of interaction dynamics. (**c**) The average and 95% confidence interval of the absolute delay between the two sides.

Fig. 3a-b show the proportion of each type of interaction dynamics across the four conditions. When the board is controlled by both hands of a single person, there is no conflict or mismatch in control plans, unlike in the dyadic condition. As such, the bimanual condition provides a natural benchmark for what high coordination looks like in this task. Compared to the dyadic condition, the bimanual condition exhibited a negligible opposed ratio, a higher aligned ratio, a lower single ratio, and a higher stationary ratio.

In both bimanual and dyadic conditions, haptic feedback led to significantly higher aligned ratios and lower single ratios. Additionally, the opposed ratio decreased significantly in the dyadic condition. This pattern mirrors the difference observed between bimanual and dyadic interactions, suggesting that haptic feedback improved coordination, particularly in the dyadic condition. We used linear mixed models to analyze the effects of haptic condition and pairing condition on each of the four types of interaction dynamics. For aligned ratio, there was a significant main effect of haptic condition (F(1,13)=22.58, p<0.001), with no significant interaction. For the opposed ratio, there was a significant interaction between haptic and pairing conditions (F(1,13)=4.92, p=0.045); post-hoc tests showed that haptic feedback significantly reduced the opposed ratio in the dyadic condition (p = 0.004), but not in the bimanual condition (p = 1). For the single ratio, haptic condition had a significant main effect (F(1,13)=65.60, p<0.001) with no interaction; post-hoc tests confirmed that haptic feedback significantly reduced the single ratio in both the bimanual (p< 0.001) and dyadic (p = 0.003) conditions. For the stationary ratio, all effects were non-significant. Full statistical results are reported in Table 1. These results confirmed that haptic feedback improved dyadic coordination.

**Table 1:** LMM and Post-hoc Tests for Interaction Dynamics in Experiment 1.

	LMM summary			Post-hoc tests	
	Main Effects: Pairing condition	Main Effects: Haptic condition	Interaction	Haptic Effect: Bimanual	Haptic Effect: Dyadic
Opposed Ratio	F(1,13)=9.411, **p=0.009**	F(1,13)=7.893, **p=0.015**	F(1,13)=4.917, **p=0.045**	Estimate=-0.001, p=1	Estimate=-0.011, **p=0.004**
Aligned Ratio	F(1,13)=4.025, p=0.066	F(1,13)=22.583, **p<0.001**	F(1,13)<0.001, p=0.992	Estimate=0.052, **p<0.001**	Estimate=0.052, **p=0.007**
Single Ratio	F(1,13)=65.604, **p<0.001**	F(1,13)=27.922, **p<0.001**	F(1,13)=0.01, p=0.921	Estimate=-0.045, **p<0.001**	Estimate=-0.043, **p=0.003**
Stationary Ratio	F(1,13)=1.995, p=0.181	F(1,13)=0.018, p=0.897	F(1,13)=0.179, p=0.679		

In aligned movements, where both sides rotated the board in the same direction, one side typically initiated the movement and the other followed after a delay. To quantify the synchronization, we measured the delay between the movement onset of the two sides for all aligned movements. The absolute delay significantly decreased in the dyadic condition when haptic feedback was provided (Fig. 3c). A linear mixed model showed significant main effects of both the haptic condition (F(1,10)=11.777, p=0.006) and the pairing condition (F(1,13)=115.380, p<0.001), but no interaction effect (F(1,10)=2.770, p=0.127). Post-hoc tests confirmed that delay was significantly reduced by haptic feedback in the dyadic condition (p=0.004), but not in the bimanual condition (p=0.236).

Overall, both interaction dynamics and delay indicate that participants coordinated more effectively when haptic feedback was provided, particularly in the dyadic condition.

### Experiment 2

In this study, as in many everyday collaborative tasks, haptic feedback arises from two main sources: partner-sourced and environment-sourced. Partner-sourced feedback is generated directly by the other person’s movement, facilitating a better understanding of their movement intent. In contrast, environment-sourced haptic feedback reflects the dynamics of the shared task, such as the movement of the ball and the board in this case, and can enhance the estimation of environmental states. While Experiment 1 demonstrated that haptic feedback improves coordination, it remained unclear whether this improvement was driven by both sources or primarily by one.

To address this, we designed Experiment 2 with five haptic conditions: Full, Without, Partner, Environment, and Unrelated. The ‘Full’ and ‘Without’ conditions were identical in design to those in Experiment 1, but now tested in a new group of participants. To isolate the effects of the two sources, we decomposed the full haptic signal into partner- and environment-sourced components, presenting only one while blocking the other, yielding the ‘Partner’ and ‘Environment’ conditions. Finally, the ‘Unrelated’ condition replayed pre-recorded force profiles that were unrelated to the current task state, testing how participants collaborate in the presence of irrelevant haptic signals (see *Methods-Haptic Conditions* for details). The ball’s weight was increased to 0.15 kg to ensure that the force feedback varied within similar ranges in all conditions except ‘Without’.

Experiment 2 also consists of three sessions, with a focus on dyadic collaboration. The first and third involved individual bimanual tasks, with one block each of the ‘Full’ and ‘Without’ haptic conditions. The second session was dyadic collaboration with 25 blocks. These blocks were organized into sets of five, with each set covering all five haptic conditions in a randomized order. An example block sequence is shown in Fig. 1e. Each block consisted of 30 successful trials. Across all participants, 9900 trials were completed successfully, while 73 trials failed because the ball rolled off the board. The failed trials were excluded from further analysis.

The bimanual sessions in Experiment 2 were intended for task familiarization, assessment of individual performance, and as a baseline for comparison with dyadic trials. Therefore, only the Full and Without conditions were included, rather than all five haptic conditions. As testing the impact of all haptic conditions on bimanual trials was not the focus of this experiment, this design ensured equal practice in the two baseline conditions before the dyadic sessions.

#### Completion Time

**Fig. 4:**
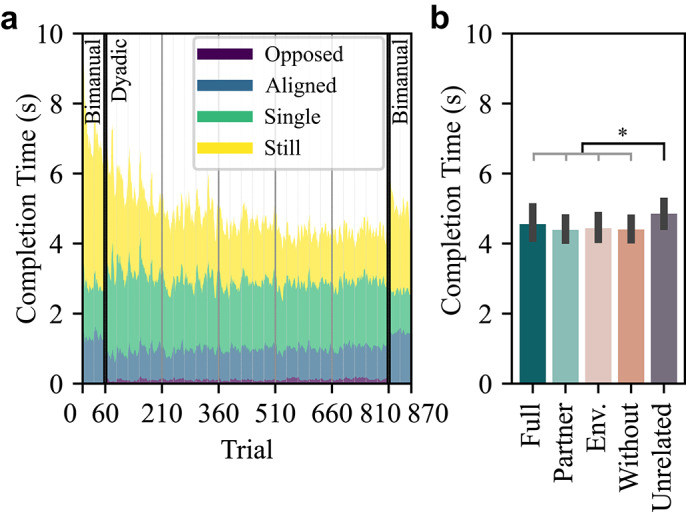
Completion time for Experiment 2. (**a**) The mean completion time across all participants as a function of trial number. Colors represent the proportion of each state of interaction dynamics. Trials 1-60 and 811-870 were performed bimanually, whereas trials 61-810 were performed dyadically. Colors indicate the average time spent performing each type of interaction dynamics. (**b**) The average completion time and 95% confidence intervals in the stable dyadic phase (blocks 6-25).

Task performance measured by completion time showed a clear improvement over the first 210 trials, before remaining fairly consistent over the rest of the experimental session (Fig 4a). Therefore, we defined Trials 211-810 (blocks 6-25) as the stable dyadic phase, excluding the initial block where each haptic condition was first introduced. In the stable dyadic phase, participants completed the task in comparable times across various haptic conditions, except for the ‘Unrelated’ haptic condition, where completion times were slightly longer (Fig 4b). A repeated measures ANOVA revealed a significant effect of the haptic condition (F(4,36)=7.73, p<0.001). Post-hoc tests showed significant differences between ‘Unrelated’ and each of the other four conditions (‘Full’: p=0.032, ‘Partner’: p<0.001, ‘Environment’: p=0.001, and ‘Without’: p<0.001). No other pairwise comparisons reached statistical significance (all p>0.61). The total rotational and translational movements of the board under each condition are presented in Supplementary Fig. S2. Phase plots for each dyad (Supplementary Figs. S3–S12) illustrate how the two participants moved relative to the ball and to each other (all in the *Supplementary Material*).

The performance plateau occurred later in Experiment 2 than in Experiment 1, likely due to differences in experimental structure. In Experiment 1, participants reached a plateau within consecutive bimanual trials under two haptic conditions, providing a stable context for adaptation. In Experiment 2, more frequent switching between bimanual and dyadic modes and among five haptic conditions (with half as many trials per block) slowed learning. The ‘Unrelated’ condition in particular likely slowed adaptation, prolonging the reaching of the plateau phase. By trial 210, all dyads had experienced every haptic condition, and a general plateau in the completion time was reached.

#### Coordination

To examine how different haptic conditions affected coordination during the dyadic tasks, we analyzed both the interaction dynamics and delay in the stable dyadic phase.

**Fig. 5:**
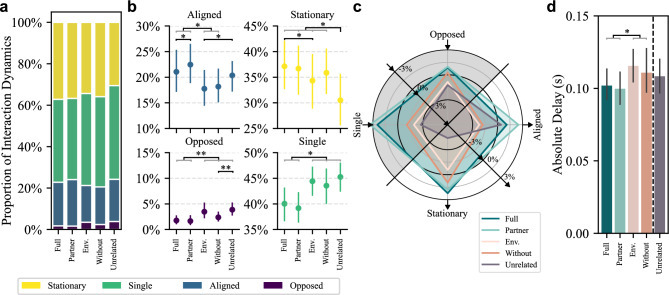
Interaction dynamics during dyadic collaboration in Experiment 2. (**a**) The proportion of each interaction dynamics category. (**b**) The average and 95% confidence interval of each category’s proportion. Conditions connected by horizontal black lines differ significantly. When multiple pairs are significant, a gray bracket is used to group them for visual clarity. Each condition in the gray bracket differs significantly from those on the opposite side of the black line. The highest p-value of all pairwise comparisons is marked by asterisks. (**c**) The relative differences in interaction dynamics across haptic conditions. The proportion of each interaction type in a haptic condition was compared with its average across all haptic conditions. For intuitive visual interpretation, the opposed and single axes were flipped (the shaded area). Thus, larger polygons indicate better overall coordination. (**d**) The absolute delay between the two participants across all dyads. Error bars represent 95% confidence interval.

We quantified the relative distribution of interaction dynamics of the dyadic collaboration across five haptic conditions (Fig. 5a, b). Repeated measures ANOVA showed a significant impact of haptic conditions on all types of interaction dynamics (all p $<0.001$). Details of the ANOVA and post-hoc test results are presented in Table 2.

The ‘Full’ and ‘Partner’ conditions demonstrated improved coordination compared to the ‘Without’ and ‘Environment’ conditions. Specifically, when comparing each of ‘Full’ and ‘Partner’ with each of ‘Without’ and ‘Environment’, the former two conditions were associated with significantly higher aligned ratios (all p$\le$0.02), lower opposed ratios (all p$\le$0.009), and lower single ratios (all p$\le$0.01). Changes in stationary ratios were generally not significant (all p $\ge$0.15, except for ‘Full’ and ‘Environment’ where p=0.01). These results replicate the findings of Experiment 1 with similar differences in the coordination between ‘Full’ and ‘Without’ conditions in a new group of participants.

The ‘Partner’ condition produced similar patterns to the ‘Full’ condition (p$\ge$0.32 for the opposed, single, and stationary ratio), with an even stronger aligned component (p=0.044). In contrast, the pattern in the ‘Environment’ condition closely resembled that in the ‘Without’ condition (all p$\ge$0.07). These results suggest that the coordination improvement observed when haptic feedback is provided is predominantly attributed to partner-sourced instead of environment-sourced haptic feedback.

Compared with the other four conditions, the ‘Unrelated’ condition exhibited a distinct pattern, with a significantly decreased stationary ratio (all p$\le$0.03), and an increase in all other types of movement ratios, that is, aligned, opposed, and single ratios. This divergence may be attributed to the disruptive nature of the irrelevant haptic feedback, potentially causing both to take control and correct each other’s actions. The high aligned ratio in ‘Unrelated’ condition therefore appears to be a byproduct of generally increased movement, which does not reflect true alignment.

**Table 2:** Summary of ANOVA and Post-hoc Tests of Experiment 2.

	ANOVA	Post-hoc tests									
	Main Effect	Full-Partner	Full-Env.	Full-Without	Full-Unrel.	Partner-Env.	Partner-Without	Partner-Unrel.	Env.-Without	Env.-Unrel.	Without-Unrel.
Opposed Ratio	F(4, 36)=22.68, **p<0.001**	p=0.32	**p=0.009**	**p<0.001**	**p=0.001**	**p=0.005**	**p=0.004**	**p**<**0.001**	p=0.07	p=0.30	**p=0.009**
Aligned Ratio	F(4, 36)=14.01, **p<0.001**	**p=0.044**	**p=0.003**	**p=0.02**	p=0.973	**p<0.001**	**p=0.004**	p=0.17	p=0.97	**p=0.02**	p=0.10
Single Ratio	F(4, 36)=22.22, **p<0.001**	p=0.60	**p<0.001**	**p=0.01**	**p=0.005**	**p<0.001**	**p=0.005**	**p=0.003**	p=0.60	p=0.60	p=0.22
Stationary Ratio	F(4, 36)=15.53, **p**<**0.001**	p=0.82	**p=0.011**	p=0.52	**p=0.002**	p=0.15	p=0.82	**p=0.011**	p=0.37	**p=0.03**	**p=0.001**

Env. and Unrel. stand for the ‘Environment’ and ‘Unrelated’ conditions, respectively.

To visualize the interaction dynamics more intuitively, we represented the interaction dynamics of each haptic condition as polygons on a radar chart with four axes, each corresponding to one type of interaction dynamic (Fig. 5c). The chart shows the relative proportions of interaction dynamics for each haptic condition compared to the average proportion across all conditions. The 0% level marks this average baseline. As discussed in Experiment 1, better coordination is indicated by higher aligned and stationary ratios, and lower opposed and single ratios. To ensure an intuitive visual interpretation, we flipped the axes for opposed and single ratios, so that extending outward indicates higher values on the aligned and stationary axes, and lower values on the opposed and single axes. Thus, a larger polygon size reflects improved interpersonal coordination.

The ‘Full’ and ‘Partner’ conditions had the largest polygons, indicating the highest overall coordination. The ‘Environment’ and ‘Without’ conditions yielded smaller polygon areas, with slightly better performance in the ‘Without’ condition. The ‘Unrelated’ condition showed a distinctly different pattern from the others. Overall, this suggests that the coordination improvement primarily stems from partner-related haptic feedback, and environment-related haptic feedback does not facilitate coordination.

The finding that partner-sourced haptic feedback is the critical component that improves coordination is further supported by the delay measures (Fig. 5d). A repeated measures ANOVA revealed a significant effect of haptic condition on the absolute delay (F(3, 27)=10.69, p<0.001). Both ‘Full’ and ‘Partner’ conditions showed significantly lower delay times than either ‘Environment’ (‘Full’ p=0.001, ‘Partner’ p<0.001) or ‘Without’ (‘Full’ p=0.03, ‘Partner’ p<0.007). There was no significant difference between ‘Full’ and ‘Partner’ (p=0.501), nor between the ‘Without’ and ‘Environment’ (p=0.317). The ‘Unrelated’ condition was not included in the statistical analysis.

### Leader-follower role distribution

Prior research has shown that humans gradually develop different roles during collaboration. This occurs not only in asymmetric scenarios, where one participant holds advantages over another, such as additional information or mechanical advantages, but also in symmetric settings, where both participants have equal access and capabilities^18,27^. A common approach to differentiate roles is categorizing participants as leaders or followers. However, the definition of leadership varies across studies and has been characterized from multiple perspectives: Leaders possess more information or superior mechanical leverage^19,21,28^; Leaders initiate joint movements, leading followers in space and time^20,29^; Leaders contribute more effort by producing more movements or forces^29–31^; Leaders engage proactively, whereas followers respond passively, often being moved by the leader’s actions^21^; Leaders produce more corrective actions^19^; Leaders set the pace to the tasks^27,32^.

**Fig. 6:**
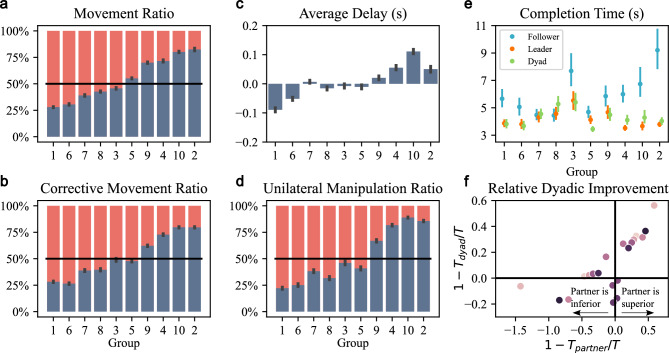
Leader-Follower role distribution (**a**) Movement ratio, showing the contributions of the left (blue) and right (red) participants to board rotation. (**b**) Corrective movement ratio, showing the proportion of corrective movements performed by the left (blue) and right (red) participants. (**c**) Average delay between participants. A positive delay indicates that the left participant initiated movement before the right, suggesting the left participant was the leader. (**d**) Unilateral manipulation ratio, showing the movement generated by the left (blue) and right (red) participants while the other participant remained stationary. (**e**) Comparison of stable completion times of individuals and dyads. Each data point represents the mean completion time with a 95% confidence interval for the final block under ‘Without’ and ‘Full’ conditions. The leader is defined as the participant with a movement ratio exceeding 50%. (**f**) Relative dyadic improvement. Each circle represents one participant, with two circles of the same color belonging to the same dyad. The x-axis ($1 - T_{\text {partner}}/T$) compares the relative performance of each participant (*T*) to their partner’s performance ($T_{\text {partner}}$) when performing the task bimanually. The y-axis ($1 - T_{\text {dyad}}/T$) indicates whether the participant’s performance improved or deteriorated when working dyadically ($T_{\text {dyad}}$). The plot shows that the worse-performing partner improved substantially when working in a team, whereas the better-performing partner’s performance changed little.

In our task, participants needed to control both the timing and magnitude of their movement. We assessed leadership through four metrics, depicted in Fig. 6, and defined in *Methods-Strategy and leader-follower relationship*. The four metrics are a) Movement Ratio: The relative proportion of each participant of the dyad to producing a rotation of the board based on a linear model of the hand movements. b) Corrective Movement Ratio: The proportion of corrective movements undertaken by each side where the cumulative corrective responses^19^ are calculated according to the absolute difference between the current position and the moving average over the last 200 ms. c) Delay: The same absolute delay metric as reported in Experiments 1 and 2, calculated as the average time lag between participants in aligned movements. d) Unilateral Manipulation Ratio: The proportion of movement between each member of the dyad, in terms of total distance across all single movements, i.e., movements performed by one participant while the other remained stationary. The leader was identified as the participant contributing more to the movement with a higher ratio or initiating it earlier. For details of the calculations, see *Methods-Strategy and leader-follower relationship*. We ordered the groups by the ascending movement ratio of the left participant and applied this order to all measures. Fig. 6a–d show a gradual shift in leadership across all four metrics, with groups exhibiting consistent leader-follower roles as identified by all measures. Notably, in Groups 3 and 5, task responsibilities were evenly distributed between the participants across all four metrics, indicating no clear leader or follower. Overall, the four measures yielded highly consistent classifications, showing a robust identification of leader-follower roles.

What determines who becomes the leader? To investigate this, we compared the individual performance of leaders and followers. Fig. 6e shows the completion times of the final block under ‘Without’ and ‘Full’ conditions for each individual (in bimanual condition) and each dyad. Leaders were determined by a movement ratio exceeding 50%. In most groups, leaders (orange) demonstrated better individual performance than followers (blue), with the exception of Groups 7 and 8, where individual performances were similar. This suggests that participants with better individual performance tend to take on the leader role. Additionally, dyadic completion times (green) closely aligned with leaders’ performance, underscoring leaders’ significant influence on task outcomes. To further assess how individuals benefited from collaboration, we quantified relative dyadic improvement using the metric proposed by Takagi et al.^16^ in Fig 6f. The x-axis ($1 - T_{\text {partner}}/T$) represents the relative completion time of each participant (*T*) compared with their partner’s performance ($T_{\text {partner}}$) in the bimanual condition, while the y-axis ($1 - T_{\text {dyad}}/T$) indicates whether performance improved or deteriorated when working dyadically ($T_{\text {dyad}}$). Participants who performed worse individually (positive x values) showed substantial improvements in the dyadic condition (positive y values). By contrast, the performance of the better-performing partners (negative x values) changed little when working dyadically. This suggests that collaboration benefited the weaker partner without disadvantaging the stronger partner, consistent with the findings of Takagi et al.^16^.

**Fig. 7:**
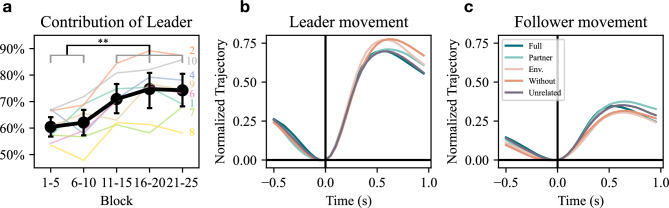
Changes in Leader-Follower Dynamics across the experiment. (**a**) Progression of the leader movement ratio over time. The thick black line represents the group mean with 95% confidence intervals, while thinner color-coded lines show individual group trends. As in the previous figures, the horizontal black line and asterisks indicate that all pairwise comparisons differ significantly between the individual members of each group. Each condition in the gray bracket differs significantly from those on the opposite side of the black line. The highest p-value of all pairwise comparisons is marked by asterisks. (**b**)-(**c**) Motion-triggered trajectory averages of the leader (**b**) and follower (**c**) in different haptic conditions. Trajectories were aligned to the board movement onset and normalized by the board angle change.

We then analyzed how leader-follower roles evolved over time by examining the movement ratio of the leaders throughout the dyadic experiment. The 25 dyadic blocks were grouped into sets of five, with each set covering all five haptic conditions. As the experiment progressed, leaders’ movement ratio increased significantly in the earlier phase, and then stabilized in the final 10 blocks (Fig. 7a). This underscores the progressive establishment of leader-follower dynamics. A repeated measures ANOVA showed a significant effect of the block number (F(4, 28)=19.36, p<0.001). Post-hoc tests showed that leaders’ contribution in early blocks (1-5 and 6-10) was significantly lower than the later blocks (11-15, 16-20, and 21-25), with all pairwise comparisons reaching statistical significance (all p$\le$ 0.002). Groups 3 and 5 were excluded due to the absence of clear leader-follower roles. These results suggest that the leader’s role strengthened over the course of the experiment.

Finally, we examined the impact of haptic condition on leader–follower role distribution. We computed the motion-triggered trajectory averages by aligning the hand trajectories to the onset of board rotation and normalizing them by the total movement required on both sides to produce the board rotation (see *Methods-Motion Triggered Trajectory Averaging* for details). The resulting trajectories reflect the temporal progression of participants’ average movement under each haptic condition. This exploratory analysis is designed to examine to what degree either the leader or follower is responsible for the upcoming motion of the board across all movements within a specific condition.

Fig. 7b and c depict the motion-triggered trajectory averages in each haptic condition for leaders and followers, with the roles determined by movement ratio. Leaders were predominantly responsible for board manipulation, contributing approximately 70% of the total movement in all conditions, which agrees with the role distribution results (Fig. 6a,c,d). While specific differences between the haptic conditions are unlikely to be statistically significant, the figures suggest towards some interesting trends. In ‘Full’ and ‘Partner’ conditions, leader contribution was slightly reduced while followers’ contribution slightly increased, compared to the ‘Without’ and ‘Environment’ conditions. This suggests a more equal distribution of leader-follower roles in the ‘Full’ and ‘Partner’ conditions, in contrast to the ‘Without’ and ‘Environment’ conditions, where the role asymmetry was enhanced. Groups 3 and 5 were again excluded due to the absence of clear leader-follower roles. Further studies are needed to determine whether such differences in the role contributions are consistent and statistically significant.

Overall these results align with our previous findings^20^ and those of Chackochan et al.^21^, which showed that leader–follower role distributions shift depending on the completeness of information. Here, we extend this conclusion by demonstrating that partner-sourced haptic feedback is the critical component driving this shift. When partner-sourced feedback is absent, mutual understanding and coordination becomes more difficult. In such cases, an enhanced leader-follower asymmetry may reduce the need for precise coordination, allowing dyads to maintain task performance despite diminished feedback.

## Discussion

Our findings demonstrate the nuanced role of haptic feedback, particularly partner-sourced feedback, in shaping collaborative coordination and role distribution. To explore this, we adapted the ball-beam system, a classic problem in control theory typically managed by a single controller^22^, into a collaborative scenario where two participants independently controlled each side of the board. This enabled redundant control, since various ways could achieve the same board angle. Therefore, participants could have a wide range of individual strategies and collaborative dynamics. Since the ball was highly responsive to board tilt, participants needed to react quickly to both environmental changes and their partner’s actions, emphasizing the need for effective communication and mutual understanding. Over time, participants adapted their strategies, modelled their partner’s behavior, and converged on patterns of effective collaboration, as reflected in the emergence of leader–follower roles. This task reflects real-world collaboration where two agents must coordinate shared control over an unstable object, such as carrying a loaded table or transporting an emergency stretcher. Modeling such dynamics offers insights into how humans coordinate in shared physical tasks and informs the design of human–robot systems for collaborative manipulation.

Haptic communication has been shown to improve task performance in many studies. A commonly used task is tracking a moving target, where performance is measured by tracking errors. Haptic communication improves tracking accuracy, with performance gains positively correlated with the stiffness of the physical coupling^17,33^. Similar improvements have been observed in tracking in force fields^34^, ankle tracking tasks^35^, and teleoperation^36^. Computational models suggest that participants can estimate their partner’s intentions through haptic feedback^16,17^. The benefits are often accompanied by increased effort associated with the physical connection^17,33^. However, a robot-mediated asymmetric connection can enhance performance without adding to the effort^37^. In our experiment, haptic feedback did not improve completion time, but did enhance interpersonal coordination. The enhanced coordination is primarily driven by partner-sourced rather than environment-sourced haptic feedback. This may be related to humans’ limited ability to allocate attention and track multiple objects visually^38,39^. When humans jointly manipulate unstable objects, such as the ball in our task, they are likely to prioritize visual attention on the object itself. In this context, partner-sourced haptic feedback may serve as a complementary information channel, compensating for limited visual attention and enhancing the overall perception of the task.

Due to the complexity and redundancy of our task, task performance was influenced by, but not solely determined by interpersonal coordination. When coordination was not ideal, dyads appeared to adopt collaboration modes that reduced coordination demands. Specifically, the leader became more dominant, while the follower became more passive^20^. This shift in role distribution may serve as a coping mechanism for the absence or degradation of haptic feedback in more complex tasks. Many studies have found that humans naturally adopt complementary roles during collaboration^18–21,27–30^. A common way to characterize these roles is by identifying participants as leaders and followers. However, different studies use different metrics to define leadership. Leaders are often described as those who initiate movement^29^, set the pace^32^, act against the mechanical coupling^21^, contribute more effort^30^, possess mechanical advantage^19^, hold exclusive information^28^, or are designated by the experimenter^27^. In our experiment, we defined the leader as the one who dominates task control. To ensure a robust assessment, we quantified dominance from four different aspects: greater contribution to task control; more initiation of movement; more corrective behavior; more unilateral control. Notably, all four metrics consistently identified the same leader-follower roles, suggesting that leadership is multifaceted yet reliably measurable through diverse indicators. We further observed that dyadic performance was largely determined by the leader’s individual ability, whereas the follower’s performance improved substantially in the dyadic setting while the leader’s performance was not strongly affected. This asymmetric benefit is consistent with the findings of Takagi et al.^16^.

We observed a gradual strengthening of leader-follower roles, with leaders becoming more dominant over time. This pattern was also reported by Takai et al.^19^. In contrast, Chackochan et al.^21^ reported that the leader-follower roles diminished with practice. This divergence may stem from differences in how leader-follower dynamics are established: In Chackochan’s study, roles emerged due to asymmetric information, which faded as partners learned each other’s private information over time. In contrast, the roles in Takai’s study were shaped by asymmetric mechanical leverage, and in ours, by different individual performance. Both factors remained stable with repetition. Chackochan et al.^21^ argued that role formation may be a suboptimal strategy to compensate for poor estimation of the partner’s intentions. Similarly, Noy et al.^27^ found that explicitly assigning roles impaired synchrony in a joint improvisation task. However, when interacting with unpredictable partners in dynamic environments, perfect partner estimation may not be feasible. In such cases, adopting and enhancing leader-follower roles can effectively reduce conflict and ensure a clear joint control strategy^19,20^. Differential game theory has been proposed as a general framework for analyzing role distribution in physical collaboration^40–42^. A range of interaction behaviors–such as co-activity, collaboration, competition, and assistance–have been successfully modeled by adjusting the cost functions and sensory feedback available to each participant. Further research is needed to improve the generalizability of these models to more complex and flexible scenarios.

While our study brings the research in haptic communication to a more complex scenario, it still does not fully capture the richness of real-world haptic interaction. The soft coupling between participants provided only low-magnitude force feedback and did not include torque. While this allowed for controlled examination of specific feedback sources, it may have constrained the emergence of more sophisticated forms of haptic communication. Future work should incorporate richer feedback modalities and explore scenarios that more closely resemble real-world collaboration. Another general challenge in haptic communication studies is that haptic feedback not only conveys information but also directly alters the task dynamics. This makes it difficult to fully disentangle whether changes in coordination arise from the information transmitted via haptic feedback or from the altered physical interaction. In our task, we believe the improved coordination is primarily driven by the informational content of haptic feedback, since the peak absolute forces were small (0.85 ± 0.39 N) and unlikely to substantially influence the partner’s movements. Nevertheless, it should be acknowledged that mechanical effects of haptic coupling may, in some contexts, contribute to improved coordination.

Our results show that humans use haptic feedback, particularly partner-sourced feedback, to improve coordination. We also demonstrated how participants establish and consolidate leader–follower roles, adapting their behavior according to different levels of partner information. These findings offer insights into the design of robotic systems that can seamlessly coordinate with humans in challenging tasks, such as service robots assisting with object transport or rehabilitation robots guiding patient movements, where mutual adaptation and coordination between humans and robots are essential. Interestingly, our results suggest that the design of haptic feedback in human–robot interaction should prioritize partner-sourced signals, which may foster even better coordination than providing full feedback.

## Conclusion

In this study, we extended haptic communication research from simple tasks to this novel task with more complex dynamics. Contrary to many prior studies^17,33–36^, we found that haptic feedback did not significantly affect overall task performance, except in the presence of unrelated haptic disturbances. However, haptic feedback enhanced the coordination between participants, as evidenced by increased alignment, reduced opposition, less unilateral manipulation, and better synchronization. This coordination enhancement was primarily driven by partner-sourced haptic feedback, whereas environment-sourced feedback had minimal effect.

Even with equal information and mechanical leverage, participants naturally developed distinct roles, with the more proficient individual becoming the leader and the less proficient adopting a follower role. Leadership consistently strengthened over time and was influenced by the availability of partner-sourced haptic feedback. Overall, our findings highlight the nuanced role of different haptic feedback sources in shaping interpersonal coordination and role distribution. This deeper understanding can directly inform the development of human-centered robotic systems designed for intuitive collaboration in real-world applications.

## Methods

### Participants

Thirty right-handed participants (22-36 years of age, 17 females, handedness assessed using the Edinburgh Inventory^43^), participated in the study after providing written informed consent. The participants engaged in the tasks both individually and in dyads. Experiment 1 involved ten participants (five dyads), while Experiment 2 included twenty participants (ten dyads). All participants were neurologically healthy and were naive to the purpose of the study. The study was approved by the Ethics Committee of the Technical University of Munich (TUM). All procedures were performed in accordance with the relevant guidelines and regulations, including the Declaration of Helsinki. Informed consent was obtained from all participants prior to their participation in the experiment. Before the experiment, participants were introduced to and familiarized with the haptic devices.

### Experimental Apparatus

The experiment was conducted in a virtual reality environment rendered by Chai3D^44^. Participants were tasked with maneuvering a ball on a frictionless board into a target area and stabilizing it there for 1.5 seconds. A trial was marked as failed if the ball rolled off the board on the left or right side. Participants used two haptic devices (Phantom Touch, 3D SYSTEMS) to control two ends of the board and perceive force feedback. Both participants can see the ball, board, and target area from the screen. Due to the indirect control of the ball and its high responsiveness to board movements, the task demanded precise control and coordination.

The task was performed under two pairing conditions: bimanual and dyadic. In the bimanual condition, participants were positioned in front of a display and manipulated the board individually using two robotic haptic devices, one in each hand (Fig. 1a). In the dyadic condition, pairs of participants, each holding a haptic device with their dominant hand, sat adjacent to each other, facing individual screens and separated by a curtain to prevent visual observation of each other’s movements; verbal communication was prohibited (Fig. 1b).

The board was connected at its center to the origin of the virtual environment by a virtual spring, which provided force feedback based on the board’s vertical deviations, thereby offering haptic information about the partner’s actions. This spring softly coupled the participants, allowing independent movements without constraining each other. The dimensions of the board-ball model are shown in Fig. 1c. The x, y, and z coordinates are oriented perpendicular to the screen to the outside, to the right, and upward, respectively. Participants could only move along the z-axis, manipulating the z-coordinates of the control points of the board via spring-damper mechanisms. The board was restricted to translations along the z-axis and rotations around the x-axis. The ball could only move along the board’s length. The target area switched between two positions with each trial.

### Virtual board-ball model

By moving their hands, participants generated forces on the board at the two control points. The forces were generated according to Equation 1.

1 $$\begin{aligned} & F_{side}=k_h \cdot (z_{side}-z_{P,side})+c_h \cdot (\dot{z}_{side}-\dot{z}_{P,side})\end{aligned}$$

2 $$\begin{aligned} & z_{P,L} = z_{board} - l \sin \theta ,\quad z_{P,R} = z_{board} + l \sin \theta \end{aligned}$$

where $side=\{L, R\}$, referring to the left or right side. $F_{\hbox {{side}}}$ is the calculated force participants applied on the board, $z_{\hbox {{side}}}$ and $z_{\hbox {{P,side}}}$ are the z coordinates of the hand and the control point on the board, respectively. The motion of the board and the ball was simulated as follows:

3 $$\begin{aligned} & F_{s}=-k_{s}~z_{board} \end{aligned}$$

4 $$\begin{aligned} & F_{L}+F_{R}-M~g-m~g~\cos ^{2}\theta +F_{s}=M\ddot{z}_{board} \end{aligned}$$

5 $$\begin{aligned} & (F_{R}-F_{L})~l~\cos \theta -m~g~x_{ball}\cos \theta =I~\ddot{\theta } \end{aligned}$$

6 $$\begin{aligned} & \ddot{x}_{ball} = -g~\sin \theta \end{aligned}$$

In these equations, $F_{\hbox {{s}}}$ is the force generated by the spring connected to the center of the board. $z_{\hbox {{board}}}$ is the z coordinate of the center of the board. $\theta$ is the board’s angle around the x-axis with positive values marking counterclockwise rotation. The values and meanings of the other parameters are summarized in Table 3. We increased the weight of the ball in Experiment 2, such that the forces in the environment and partner haptic conditions are within similar ranges.

Our model simplified the task dynamics by assuming the normal force between the board and the ball was always $mg\cos \theta$. This guaranteed that the ball always stayed in contact with the board, preventing it from being “thrown off” during rapid movements. Since participants generated only subtle movements within small ranges, this assumption closely approximated the realistic interaction force, with a deviation of $0.025\pm 0.015$ N. We calculated the relative ball position using the above equations, which was then used for visualization.

**Table 3:** Parameters of the virtual board-ball model.

Symbol	Parameter	Unit	Value
*M*	Weight of the board	*kg*	0.01
*m*	Weight of the ball (Exp. 1)	*kg*	0.05
*m*	Weight of the ball (Exp. 2)	*kg*	0.15
*g*	Gravity acceleration	$m/s^2$	9.81
$k_h$	Force input stiffness	*N*/*m*	200
$c_h$	Force input damper	*Ns*/*m*	2
$k_{s}$	Stiffness of the spring	*N*/*m*	140
*l*	Distance between the control point and the center of the board	*m*	0.25
*I*	Moment of inertia of the board	$kg \cdot m^2$	0.0004

### Haptic conditions

This study primarily investigates the role of haptic feedback in facilitating physical collaboration. In our experiment, as well as in many daily activities, haptic feedback mainly originates from two sources: The partner’s movement and the environmental changes. To examine the impact of each haptic feedback source, we designed the following haptic conditions:

- **Without**: Participants do not receive any force in the vertical direction, i.e., $F_L^{FB}=F_R^{FB}=0$.
- **Full**: Participants receive the same amount of force as they exerted on the board, i.e., $F_L^{FB}=-F_L, F_R^{FB}=-F_R.$
- **Partner**: Participants only receive the haptic feedback derived from participants’ movements. In our design, this is the same as assuming the board and ball are weightless, such that participants only receive the forces from the central spring, i.e., $F_L^{FB}=F_R^{FB}=\frac{1}{2}F_s$.
- **Environment**: Participants received haptic feedback exclusively from environmental changes. Since partner-sourced forces primarily originate from the central spring, the environment-sourced forces are calculated by assuming the central spring is non-existent, i.e., $F_L^{FB}=-F_L-\frac{1}{2}F_s, \quad F_R^{FB}=-F_R-\frac{1}{2}F_s$.
- **Unrelated**: Participants experienced forces unrelated to the immediate experimental conditions. We obtained 30 force profiles from pilot experiments conducted under identical settings, where participants experienced the ‘Full’ haptic condition. These 30 force profiles will be presented to participants across 30 trials in a random order. Should a trial extend beyond the duration of a force profile, the profile will be replayed, with a ramp between the start and end, to maintain signal continuity. Consequently, while the force pattern (frequency, amplitude, etc.) perceived by the participants was similar to that of the present experiment, it was unrelated to the real-time events of the current experiment.

Note that the partner-sourced and environment-sourced haptic feedback sum up to the full haptic feedback. Unrelated haptic feedback was included to examine the effect of external disturbances. The overall absolute force feedback was similar across all four haptic feedback types: Full ($0.48 \pm 0.35$ N), Partner ($0.55 \pm 0.39$ N), Environment ($0.37 \pm 0.16$ N), and Unrelated ($0.55 \pm 0.43$ N), reported as mean force ± standard deviation.

### Experimental paradigm

Two experiments were conducted in this study: Experiment 1 examines two haptic conditions (‘Full’ and ‘Without’) across three sessions (Fig. 1d). The first and third sessions involve individual bimanual manipulation, with participants completing four blocks in the first session and two in the third. The second session examines dyadic collaboration, with six experimental blocks. Each block consists of 60 successful trials under one haptic condition. The ‘Full’ and ‘Without’ haptic conditions alternate between successive blocks, with the order being randomized for each participant or dyad.

Building on the findings from Experiment 1, Experiment 2 expands the range of haptic conditions: ‘Without’, ‘Full’, ‘Partner’, ‘Environment’, and ‘Unrelated’, with a greater emphasis on dyadic collaboration (Fig. 1e). It consists of three sessions. The first and third involve individual bimanual tasks, with one block each of ‘Full’ and ‘Without’ haptic conditions. The second session is dedicated to dyadic collaboration, where dyads work through 25 blocks. These blocks are organized into sets of five, each set covering all five haptic conditions in a randomized order. Each new set features a different randomized order. Each block consisted of 30 trials.

Each trial started with the ball fixed at the center of one target area. Auditory and visual cues were given to signal the onset of a new trial. Participants manipulated the two sides of the board to slide the ball across and stabilize it in the opposite target area. A trial was completed when the ball remained in the target area for 1.5 seconds. Participants were then required to keep the board horizontal for one second until the next trial started. Right before the start of the next trial, the target area switched to the opposite side. If the ball fell off the edge of the board, the trial would be marked as failed and was repeated. The trial completion time was displayed after each trial, and participants were encouraged to complete them as quickly as possible.

### Data analysis

Kinematic and dynamic data were sampled at 1000 Hz and low-pass filtered with a tenth-order Butterworth filter with a 20 Hz cutoff frequency applied in dual-pass mode to achieve zero phase lag while removing high-frequency noise. Data analysis was performed with Python 3.8.

#### Completion time

Completion time was defined as the time from the beginning of each trial to when the ball had stayed within the target area for 1.5 seconds.

#### Movement segmentation

**Fig. 8:**
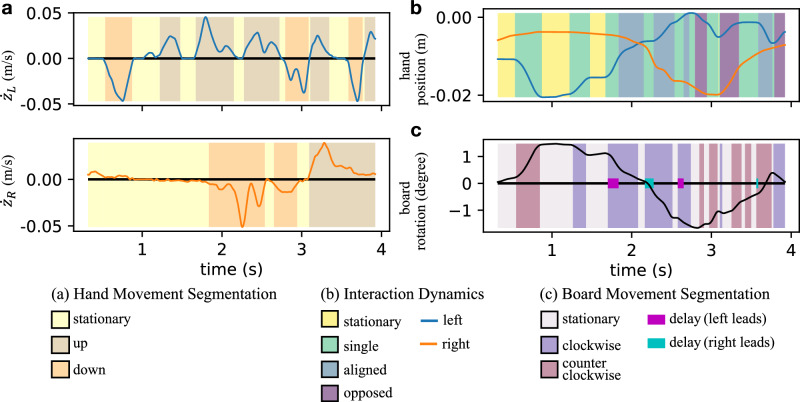
Segmentation example of a representative trial. (**a**) Velocity profile and HMM hand movement segmentation for the left and right sides, respectively. (**b**) Left and right-hand trajectories and the interaction dynamics. (**c**) Angle of the board, the HMM board rotation segmentation, and the extracted delay between the agents.

We observed that participants typically controlled the board through discrete movements rather than continuously. To identify the start and end of these discrete movements, we employed unsupervised segmentation of the velocity profiles for the hand and the board using hidden Markov models (HMM)^45^. For hand movement segmentation, we trained an HMM to classify the hand movement into one of three states: moving down, stationary, or moving up (Fig. 8a). Similarly, we trained an HMM for the rotation of the board. This HMM used the angular velocity profile of the board to predict if it is rotating clockwise, stationary, or rotating counterclockwise (Fig. 8c). Compared to simple thresholding, HMM-based segmentation is generally more robust to noise, as it incorporates temporal continuity and probabilistic modeling of state transitions.

#### Coordination

In this work, coordination refers to the ability to work together effectively and efficiently toward a common goal. Specifically, it involves the synchronization of actions, with both agents striving to rotate the board in the same direction simultaneously. We quantify coordination through measures of interaction dynamics and delay.

**Interaction dynamics** Based on the results of hand movement segmentation, each side could only be in one of three states at each time point: Moving up, moving down, or stationary. Consequently, the two sides have nine possible combinations of movement states. We grouped these nine combinations into four categories of movement dynamics:

- **Aligned**: the two sides move in opposite z-axis directions, aligned in rotating the board in the same direction.
- **Opposed**: both sides move in the same z-axis direction, opposing each other in rotating the board.
- **Single**: one side moves while the other side remains stationary.
- **Stationary**: both sides are stationary.

In our previous work^20^, ‘aligned’ movements were termed ‘cooperative’ and ‘opposed’ movements were termed ‘competitive.’ However, here we have modified the terminology to avoid potential goal-level implications which might vary depending on the task provided to participants and instead focus on the instantaneous control of the board. The definitions are otherwise identical to our previous work. An example of interaction dynamics is depicted in Fig. 8b. The proportion of each interaction category was calculated as the total time spent on each category divided by the completion time.

**Delay** When both sides try to rotate the board in the same direction, it is typical for one side to initiate the movement and the other to follow, resulting in a time difference. For each board movement segment, the delay is determined as the time difference between the onset of board rotation and the onset of aligned movement (see Fig. 8c). The onset of movements was determined by the result of the hand and board movement segmentation (detailed in *Methods-Movment segmentation*). A positive delay indicates that the right side moves ahead of the left side, while a negative delay indicates the reverse. This analysis focused on the board movement segments classified as aligned.

#### Strategy and leader-follower relationship

Previous studies indicated that collaborating participants often establish distinct roles, even in symmetric experiment settings where participants had equal access to information and mechanical leverage^18,20^. Roles are defined differently depending on the task. A common approach categorizes participants as ‘leaders’ and ‘followers’. However, different studies adopt various metrics to quantify leadership. In this work, a leader is defined as the participant who dominates in the task control. To quantify leadership, we employed four metrics: the movement ratio, which measures the relative contribution to board manipulation; the average delay, assessing which side initiates movements more often; the corrective movement ratio, evaluating the ratio of corrective actions generated by each side; and the single movement ratio, determining the proportion of individual manipulation of the board.

**Movement ratio** In this task, participants manipulate the board to control the ball indirectly. To simplify the interaction between the participants and the board, similar to our previous work^20^, we used a linear model (Eq. 7-8) to map the board angles to hand positions. The theoretical and empirical justification for applying a linear model is presented in Supplementary Methods and Supplementary Fig. S1.

7 $$\begin{aligned} z_{L}&=-k_{\theta -L} * \theta + b_{\theta -L} \end{aligned}$$

8 $$\begin{aligned} z_{R}&=k_{\theta -R} * \theta + b_{\theta -R} \end{aligned}$$

where $k_{{\theta \hbox {-L}}}$, $k_{{\theta \hbox {-R}}}$, $b_{{\theta \hbox {-L}}}$ and $b_{{\theta \hbox {-R}}}$ are the gains and intersection points of the participants on the left and right, respectively. The ratio between $k_{{\theta \hbox {-L}}}$ and $k_{{\theta \hbox {-R}}}$ indicates the relative contribution of the two agents in controlling the board’s motion.

9 $$\begin{aligned} p_{\theta -L}&=k_{\theta -L} / (k_{\theta -L} + k_{\theta -R}) \end{aligned}$$

10 $$\begin{aligned} p_{\theta -R}&=k_{\theta -R} / (k_{\theta -L} + k_{\theta -R}) \end{aligned}$$

where $p_{{\theta \hbox {-L}}}$, $p_{{\theta \hbox {-R}}}$ are the proportions of the angle-hand gain. Using this model, we calculated the movement ratio as the proportion of movement on each side to achieve a specific board angle. An agent whose contribution exceeds 50% is defined as a leader since they play a more dominant role in manipulating the board angle. As shown in Fig. 8, participants typically made discrete movements to manipulate the board^46^. Therefore, not every hand position at each time step reflects the participants’ desired position. We propose that participants reach their desired position when the board has just stopped rotating. We used the board angle ($\theta$) and hand position ($z_{\hbox {{L}}}$, $z_{\hbox {{R}}}$) at the end of each board movement segment to fit the linear model.

**Average delay** We used the average delay (calculation detailed in *Methods-Delay*) to quantify the initiation of movements. A positive average delay indicates that the right side often moves ahead of the left side, i.e., the right side is the leader, while a negative average delay indicates the reverse.

**Unilateral manipulation ratio** We propose that leaders should have a larger individual influence on the board dynamics. During single movements, when only one participant is manipulating and the other one is stationary, we calculated each participant’s movement distance. The unilateral manipulation ratio was calculated as the ratio of the movement distance on each side. The leaders should have a higher ratio compared to the followers.

**Corrective movement ratio** Takai et al. suggested that leaders generate more corrective behavior than followers^19^. The corrective movements reflect the active corrections and immediate adjustments to maintain control. It is quantified as the absolute difference between the current position and its moving average over the past 200 ms. We calculated the cumulative corrective movements of each participant per trial as follows:

11 $$\begin{aligned}&C_{side} = \frac{1}{N} \sum _{t=1}^{N} \left| z_{side}(t) - \frac{1}{min(k,t)} \sum _{j=0}^{min(k,t)-1} z_{side}(t-j) \right| \end{aligned}$$

12 $$\begin{aligned}&CR_{side} = \frac{C_{side}}{C_L+C_R} \end{aligned}$$

where $C_{\hbox {{side}}}$ and $CR_{side}$ denote the cumulative corrective movement and the corrective movement ratio on each side, *N* is the total number of time points in the trial, and *k* is the time window set to 200 ms. Similar to the unilateral manipulation ratio, leaders should have a higher corrective movement ratio than followers.

**Motion Triggered Trajectory Averaging** To analyze the general pattern in the participants’ movement trajectories amid variability, we developed a method inspired by spike-triggered averaging^47^, where we aligned all trajectories by the onset of each board rotation. Based on the result of the board movement segmentation, we defined a time window from 0.5 seconds before to 1 second after the board movement onset, with the onset aligned to 0 seconds in the timeline. The trajectory of each participant within this time window was shifted such that the hand position was zero at the onset of board movement. The trajectory was then normalized by the ‘required total movement’, which is the total movement on both sides necessary for the angle change. The normalized trajectories show the proportional contributions of each participant to the board rotation. We combined data from all participants and trials, then calculated the aligned average trajectories under each haptic condition for leaders and followers, respectively. Large and small angle changes contribute equally to the average trajectory due to normalization. The aligned average trajectory was calculated as follows:

13 $$\begin{aligned}&L_{\text {req}}^{i} = 2l\big (\sin \theta _{\text {end}}^{i} - \sin \theta _{\text {start}}^{i}\big ),\end{aligned}$$

14 $$\begin{aligned}&\tilde{Tr}_{L}^{i}(t) = -\frac{Tr_{L}^{i}(t)}{L_{\text {req}}^{i}}, \qquad \tilde{Tr}_{R}^{i}(t) = \frac{Tr_{R}^{i}(t)}{L_{\text {req}}^{i}},\end{aligned}$$

15 $$\begin{aligned}&NT(t) = \frac{1}{N_{\text {segments}}}\sum _{i\in S} \tilde{Tr}_{\text {side}}^{i}(t). \end{aligned}$$

where *i* indexes the *i*-th board movement segment. $L_{\hbox {{req}}}^{i}$ is the required total movement on both sides for the *i*-th segment. $\theta _{\hbox {{start}}}^{i}$ and $\theta _{\hbox {{end}}}^{i}$ are the board angles at the start and end of the *i*-th segment. ${Tr}_{\hbox {{L}}}^{i}$ and ${Tr}_{\hbox {{R}}}^{i}$ are the aligned trajectories for the left and right sides, respectively. The trajectories are aligned in time to the onset of the segment and in position to the hand position at the onset. $\tilde{Tr}_{L}^{i}$ and $\tilde{Tr}_{R}^{i}$ are the trajectories normalized by $L_{\hbox {{req}}}^{i}$ for the left and right sides, respectively. They represent the proportion of movement that each side contributed to rotating the board. For directional alignment, the trajectories on the left side were flipped. *NT* is the average normalized trajectory. *S* is the set of all segments meeting the conditions (e.g. Leader’s movement in the ‘Full’ haptic condition). $N_{segments}$ is the number of segments within *S*. We calculated *NT* for leaders and followers in each haptic condition.

### Statistical analyses

In Experiment 1, linear mixed models were applied, with the behavioral metrics as the dependent variables. Pairing condition (Bimanual/Dyadic) and haptic condition (Full/Without) served as fixed effects. Subjects were treated as random effects, incorporating both random intercepts and slopes for the haptic condition. Data from all trials within the specified conditions were included. Degrees of freedom were rounded to the nearest whole number as per Satterthwaite’s approximation. Post-hoc comparisons were conducted using contrasts on the estimated marginal means with Holm corrections. The ‘Full’ haptic condition was coded as 1, and the ‘Without’ haptic condition as -1.

In Experiment 2, the analysis was restricted to the dyadic blocks, using repeated measures ANOVA to evaluate the difference across five haptic conditions (Full, Partner, Environment, Without, and Unrelated). The average value across all trials within the specified conditions was included. Post-hoc comparisons were conducted using paired t-tests with Holm corrections.

Different statistical analyses were chosen for the two experiments due to differences in design. In Experiment 1, the same individuals performed both bimanual and dyadic trials, resulting in a partially crossed structure that cannot be well handled by rmANOVA. Linear mixed-effects models allowed us to properly account for this dependency. In Experiment 2, we only compared dyadic performance across haptic conditions. For this simpler within-subject design, we used rmANOVA, which is widely used in the field. We note that linear mixed-effects models could also be applied to Experiment 2 and would yield equivalent conclusions.

Analyses were performed using JASP version 0.18.3.0. The results are considered statistically significant when p-values are less than 0.05. Statistical comparisons are based on all pairwise tests between individual conditions. In the figures, black horizontal lines indicate significant differences. When several pairs are significant, we use a gray bracket to group together conditions that all differ significantly from those on the other side of the black horizontal line. The gray bracket is only for visual clarity, and it does not indicate a collapsed statistical test. The highest p-value of all pairs is denoted by * (p < 0.05), ** (p < 0.01), and *** (p < 0.001) in the figures.

## Supplementary Information

Supplementary Information.

## Data Availability

The data used in this study are available from the corresponding authors, Y.L. and D.F., upon reasonable request.
