# Supplementary material for: Partner-sourced haptic feedback rather than environmental inputs drives coordination improvement in human dyadic collaboration
Source: Sci Rep. 2025 Nov 18;15:40347. doi: 10.1038/s41598-025-27258-5 (PMC12627734; doi:10.1038/s41598-025-27258-5)
Supplement: Supplementary file 1 — Supplementary Information. [file 41598_2025_27258_MOESM1_ESM.pdf]

Partner-sourced haptic feedback rather than environmental inputs drives coordination improvement in human dyadic collaboration

Yiming Liu, Raz Leib, William Dudley, Ali Shafti, A. Aldo Faisal and David W. Franklin

Supplementary Information

Supplementary Methods

Justification of using a linear model to quantify the movement ratio.

To quantify the relative contribution of each side in controlling the board, we used a linear model. Supplementary Figure S1 shows one block of a typical dyad, where the hand position is plotted as a function of board angle, demonstrating a clear linear trend.

This linearity also follows directly from the physical model of the board. The vertical displacement difference between the two sides is $z_{P,R} - z_{P,L} = 2l$. Since participants rotated the board within a small range (max 2.38±1.02 deg), we can approximate$\theta \approx \theta$. So, to achieve a desired board angle, the two sides need to reach a vertical difference proportional to the board angle. Because the board's vertical position was also within a small range (0.010±0.005 m), we assumed that participants manipulated the board around a desired vertical position z_{desire}.

If the two sides contribute to the total movement by certain movement ratiosMR_L and MR_R ($MR_L + MR_R = 1$), then $z_L \approx z_{desire} - MR_L \cdot 2l$ and $z_R \approx z_{desire} + MR_R \cdot 2l$. This confirms that a linear model is appropriate for estimating the movement ratio.

An alternative, perhaps more intuitive way to quantify the movement ratio would be based on the total hand movement distance. However, this method is less robust because a participant could move constantly within a small range, accumulating substantial distance while contributing little to board rotation. In our linear model, such a participant would have a small slope and a small movement ratio. This makes the linear model more robust than distance-based alternatives.

Supplementary Figures

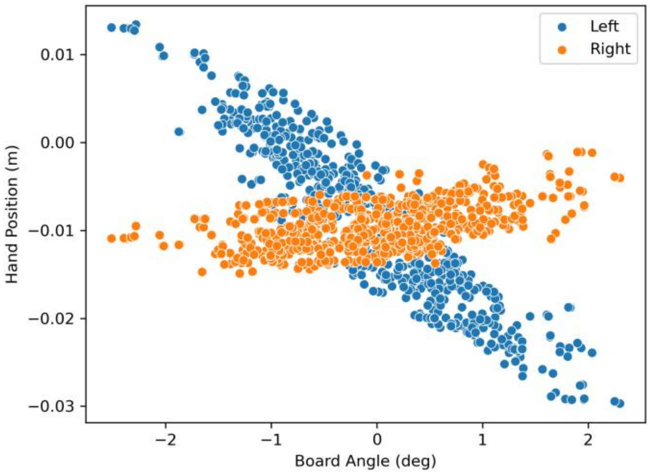

Supplementary Figure S1. Scatter plot of the hand positions on both sides as a function of the board angle. The linear relationship shows how changes in hand position relate to board rotation. The slope reflects the relative adjustment each side makes to achieve a given angle, capturing their contribution to the movement.

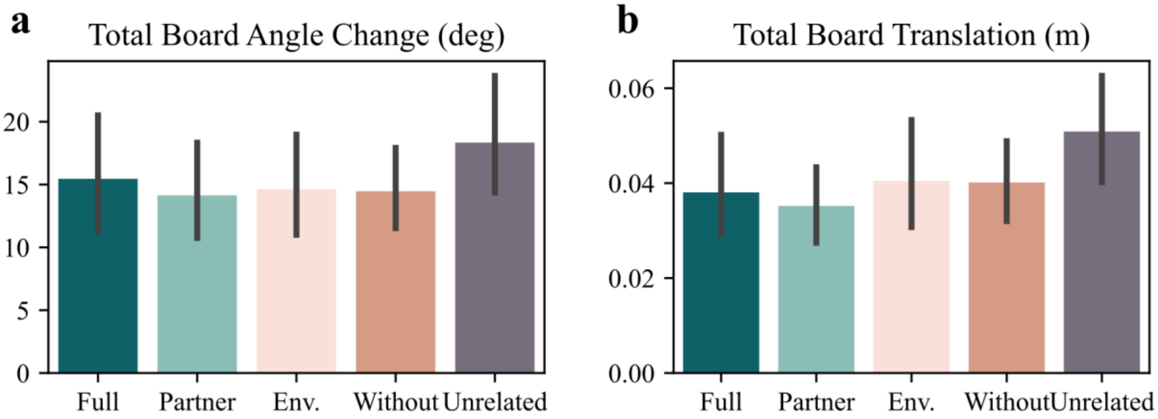

Supplementary Figure S2. Total rotational and translational movement of the board under each haptic condition in the stable dyadic phase (Block 6-25) in Experiment 2. a) The integral of rotation around the center of the board. b) The integral of vertical movements of the center of the board.

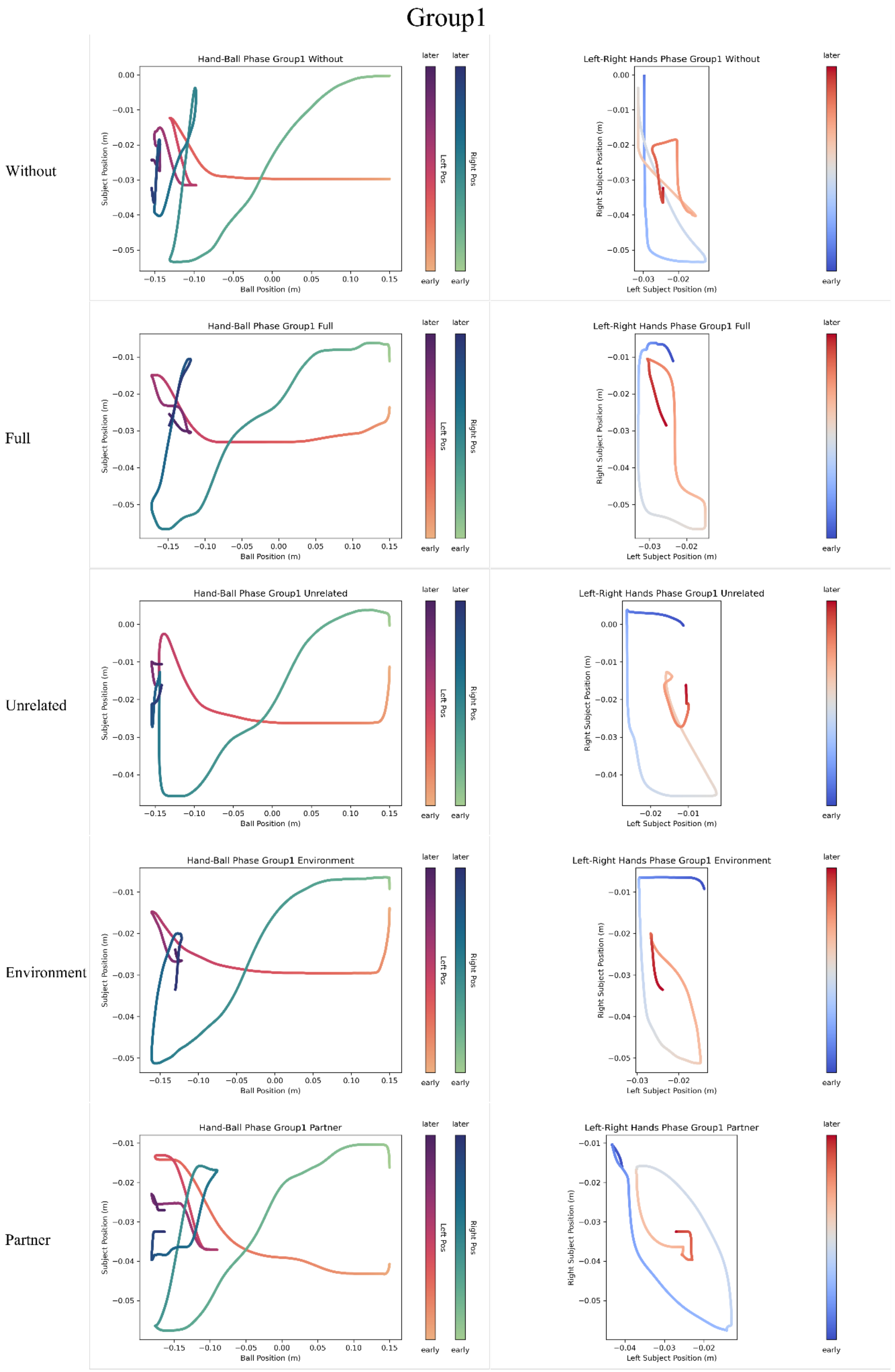

Supplementary Figure S3. Phase plots for Group 1. These plots display the relationship between hand and ball positions (Hand-Ball plots) and the relationship between the hand movements of both participants (Left-Right Hand plots). Each row represents a different haptic condition, with data taken from the final trial of each haptic condition for each dyad in the dyadic condition. Diverging colors indicate progression from early to late in the trial.

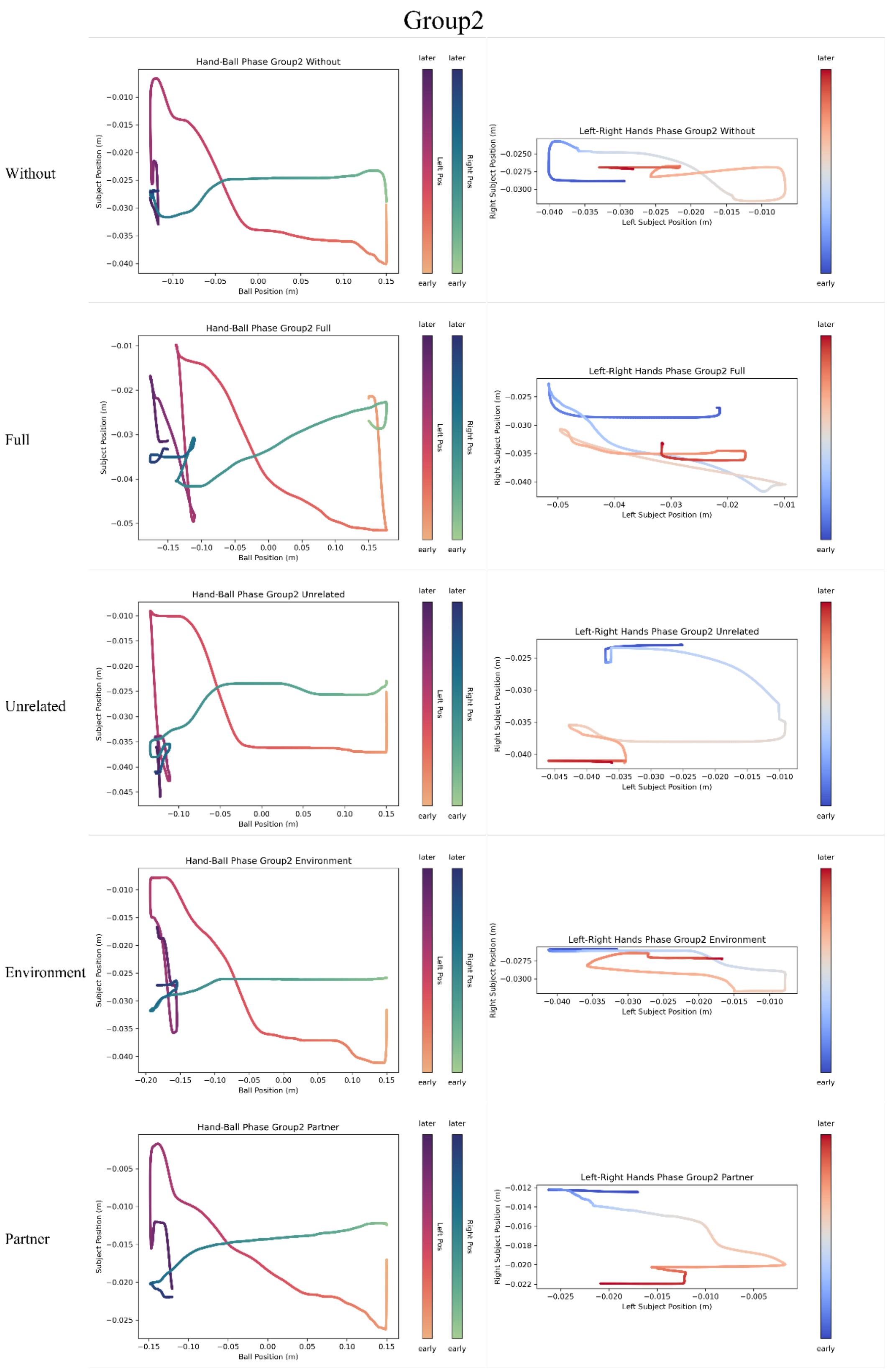

Supplementary Figure S4. Phase plots for Group 2. These plots display the relationship between hand and ball positions (Hand-Ball plots) and the relationship between the hand movements of both participants (Left-Right Hand plots). Each row represents a different haptic condition, with data taken from the final trial of each haptic condition for each dyad in the dyadic condition. Diverging colors indicate progression from early to late in the trial.

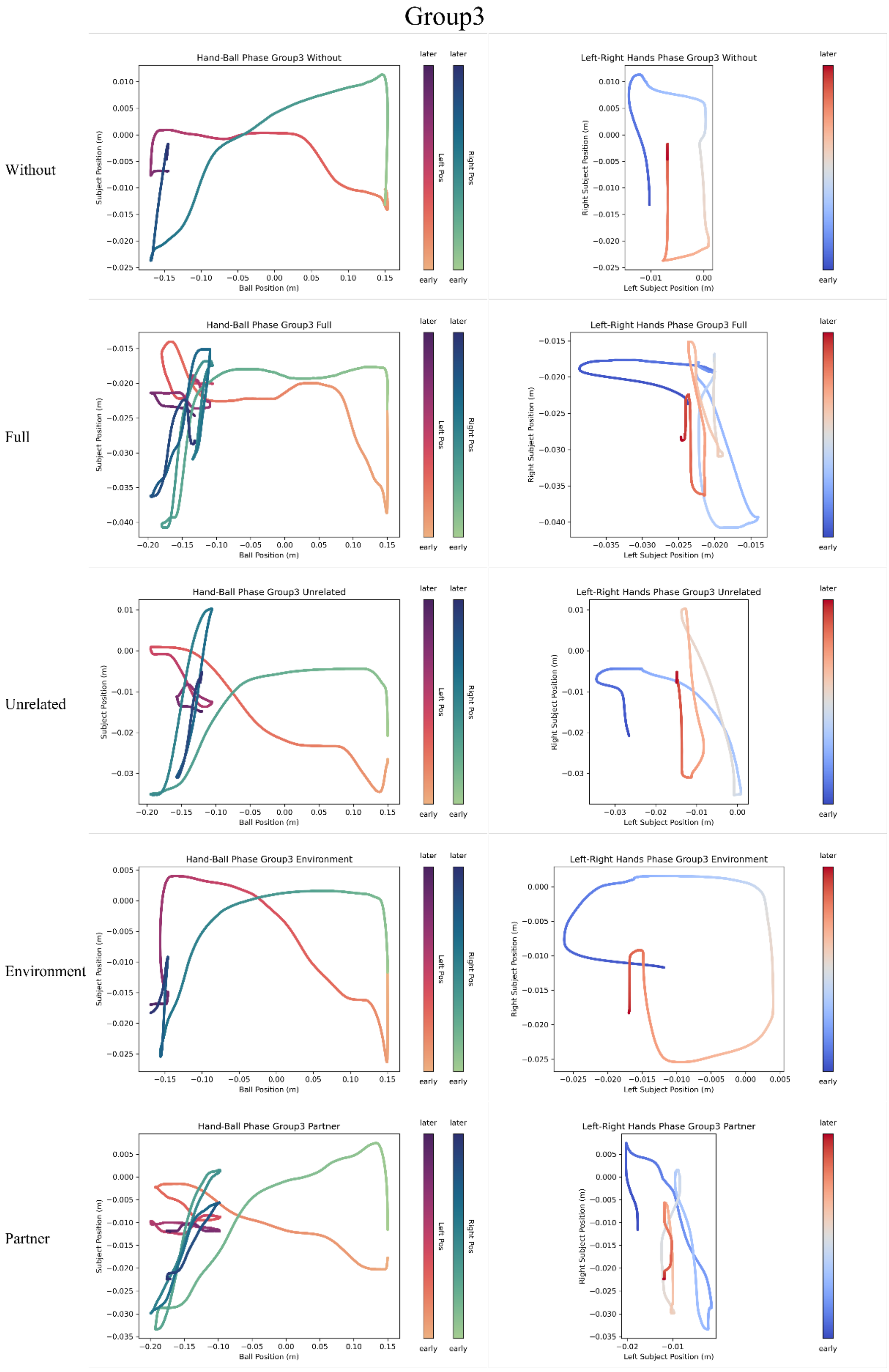

Supplementary Figure S5. Phase plots for Group 3. These plots display the relationship between hand and ball positions (Hand-Ball plots) and the relationship between the hand movements of both participants (Left-Right Hand plots). Each row represents a different haptic condition, with data taken from the final trial of each haptic condition for each dyad in the dyadic condition. Diverging colors indicate progression from early to late in the trial.

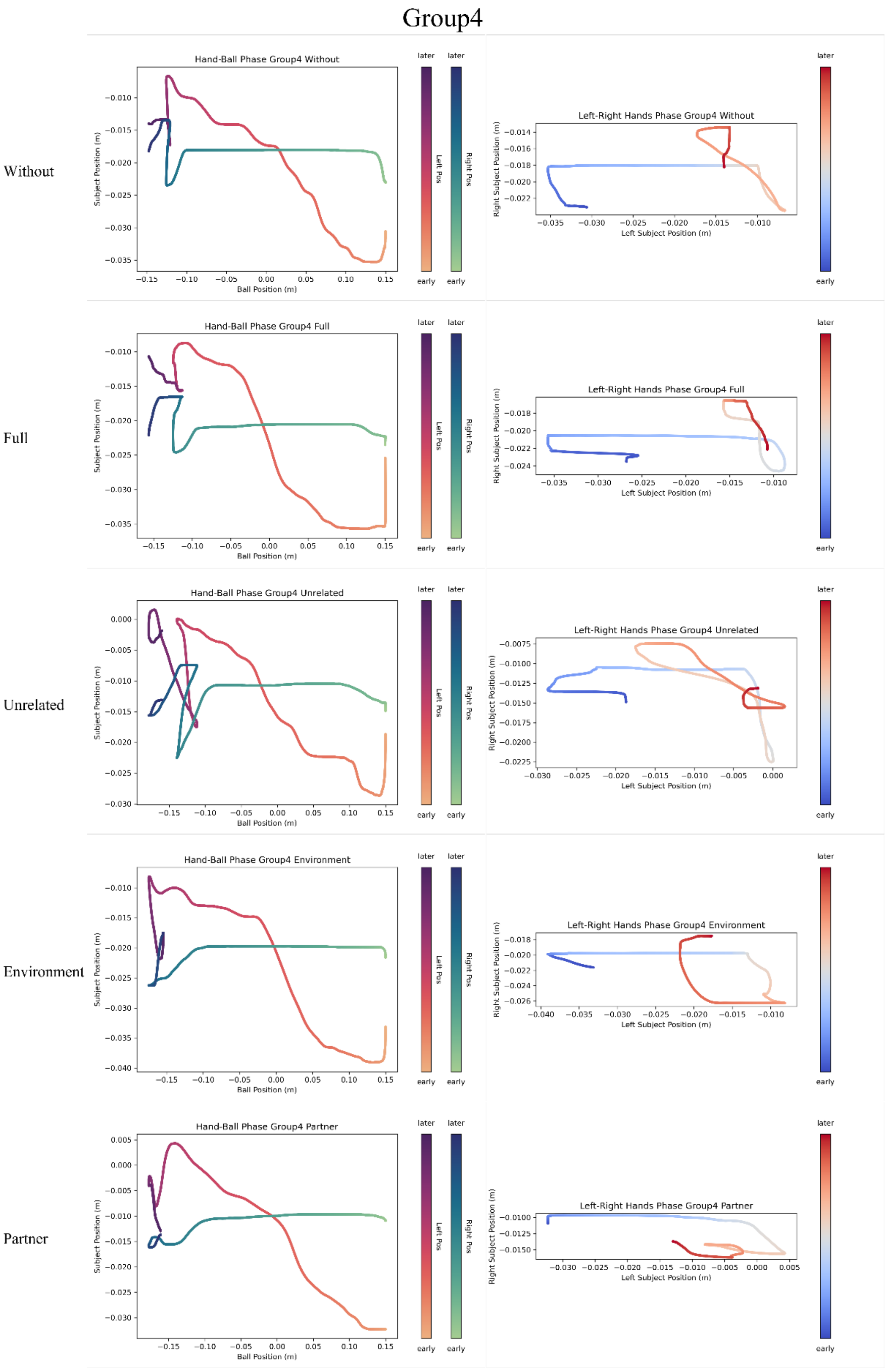

Supplementary Figure S6. Phase plots for Group 4. These plots display the relationship between hand and ball positions (Hand-Ball plots) and the relationship between the hand movements of both participants (Left-Right Hand plots). Each row represents a different haptic condition, with data taken from the final trial of each haptic condition for each dyad in the dyadic condition. Diverging colors indicate progression from early to late in the trial.

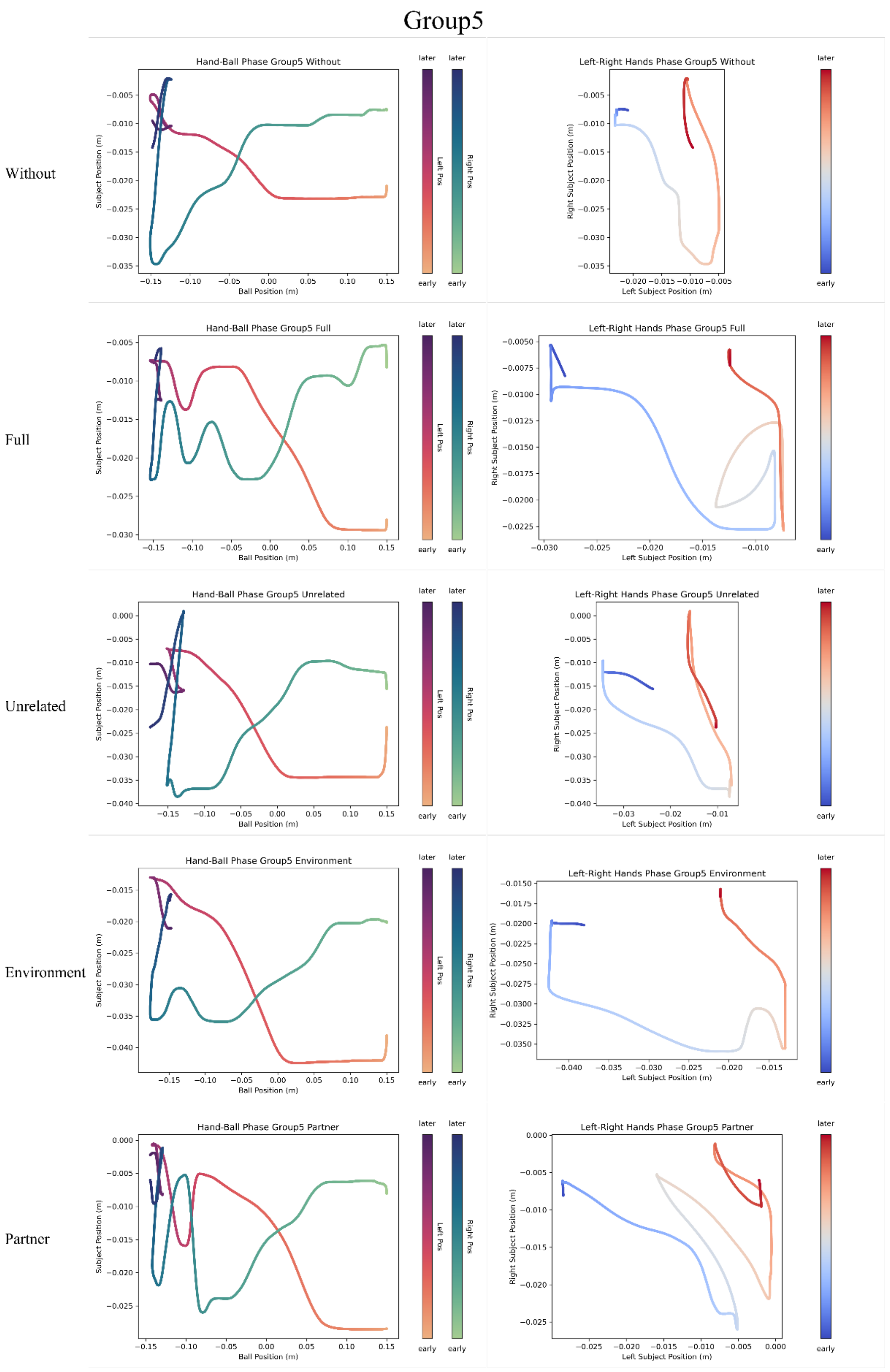

Supplementary Figure S7. Phase plots for Group 5. These plots display the relationship between hand and ball positions (Hand-Ball plots) and the relationship between the hand movements of both participants (Left-Right Hand plots). Each row represents a different haptic condition, with data taken from the final trial of each haptic condition for each dyad in the dyadic condition. Diverging colors indicate progression from early to late in the trial.

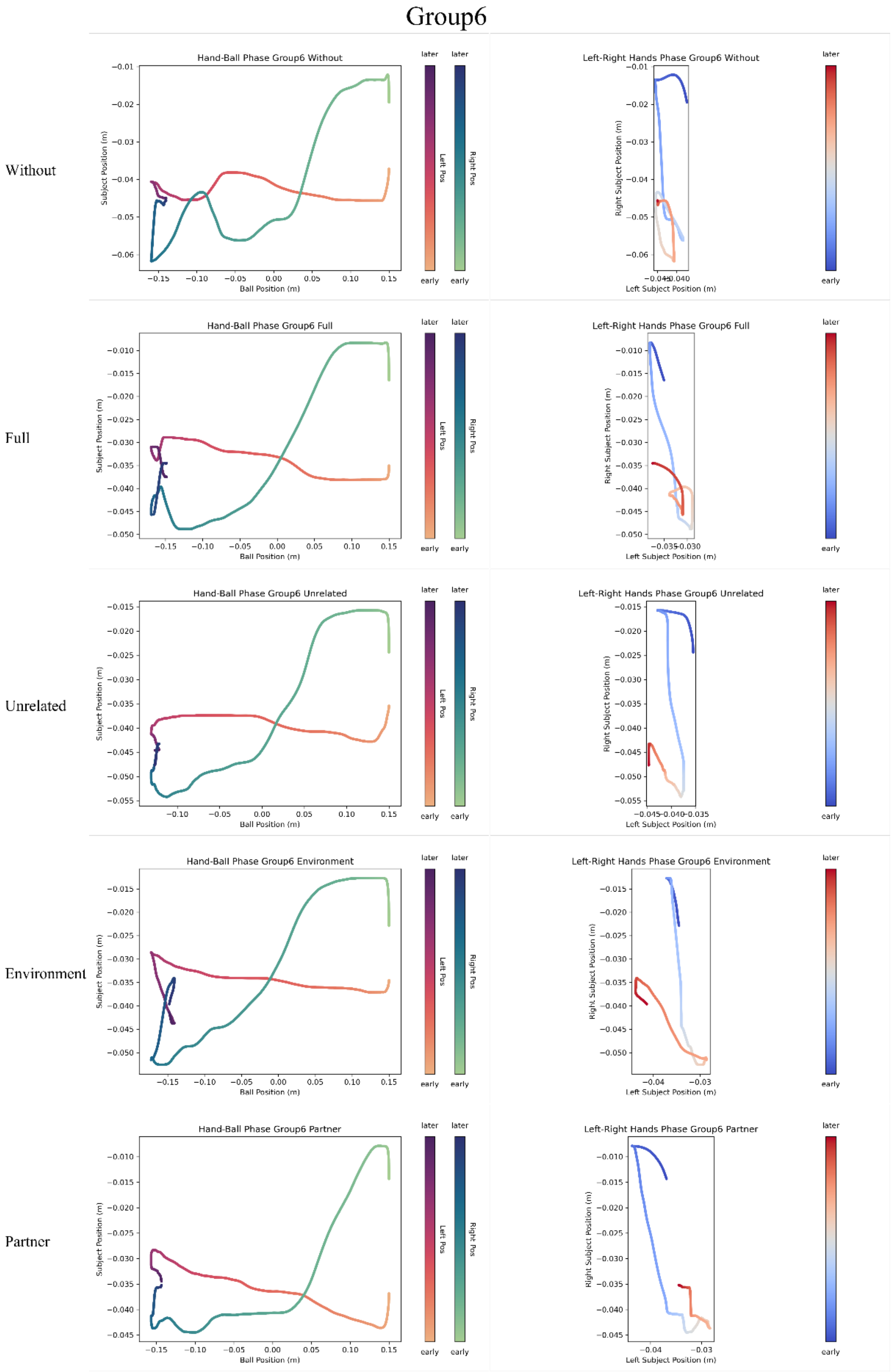

Supplementary Figure S8. Phase plots for Group 6. These plots display the relationship between hand and ball positions (Hand-Ball plots) and the relationship between the hand movements of both participants (Left-Right Hand plots). Each row represents a different haptic condition, with data taken from the final trial of each haptic condition for each dyad in the dyadic condition. Diverging colors indicate progression from early to late in the trial.

Group7

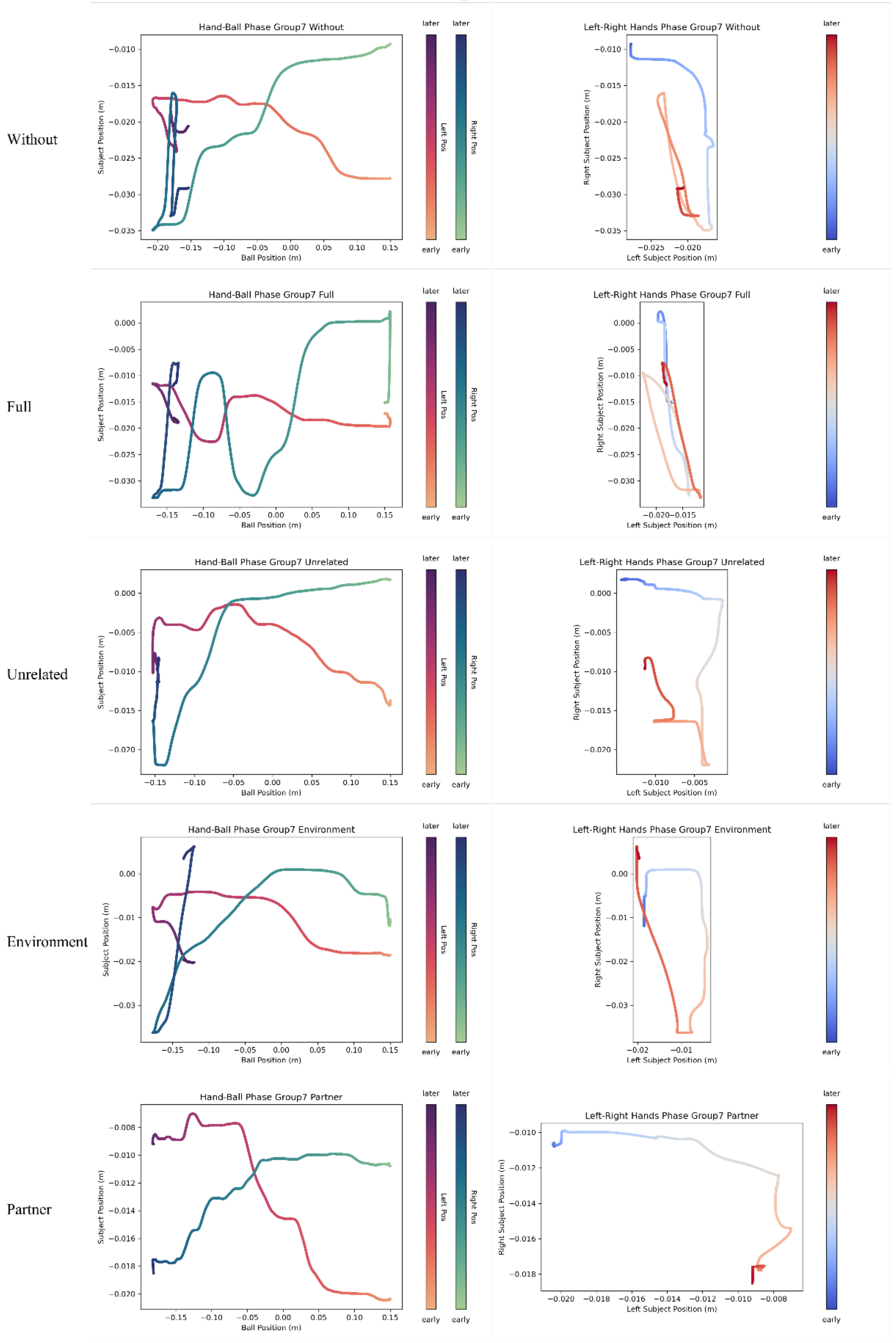

Supplementary Figure S9. Phase plots for Group 7. These plots display the relationship between hand and ball positions (Hand-Ball plots) and the relationship between the hand movements of both participants (Left-Right Hand plots). Each row represents a different haptic condition, with data taken from the final trial of each haptic condition for each dyad in the dyadic condition. Diverging colors indicate progression from early to late in the trial.

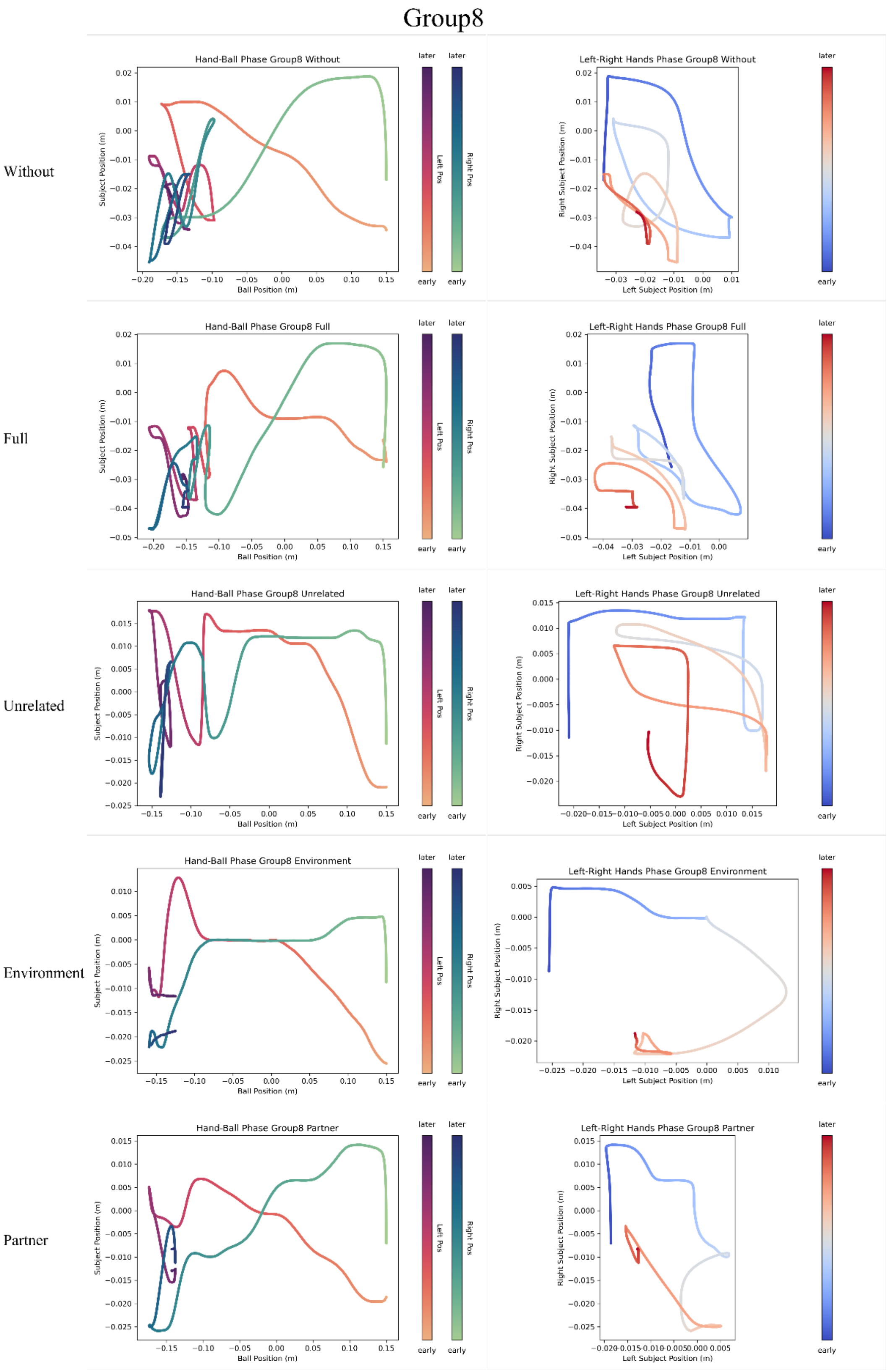

Supplementary Figure S10. Phase plots for Group 8. These plots display the relationship between hand and ball positions (Hand-Ball plots) and the relationship between the hand movements of both participants (Left-Right Hand plots). Each row represents a different haptic condition, with data taken from the final trial of each haptic condition for each dyad in the dyadic condition. Diverging colors indicate progression from early to late in the trial.

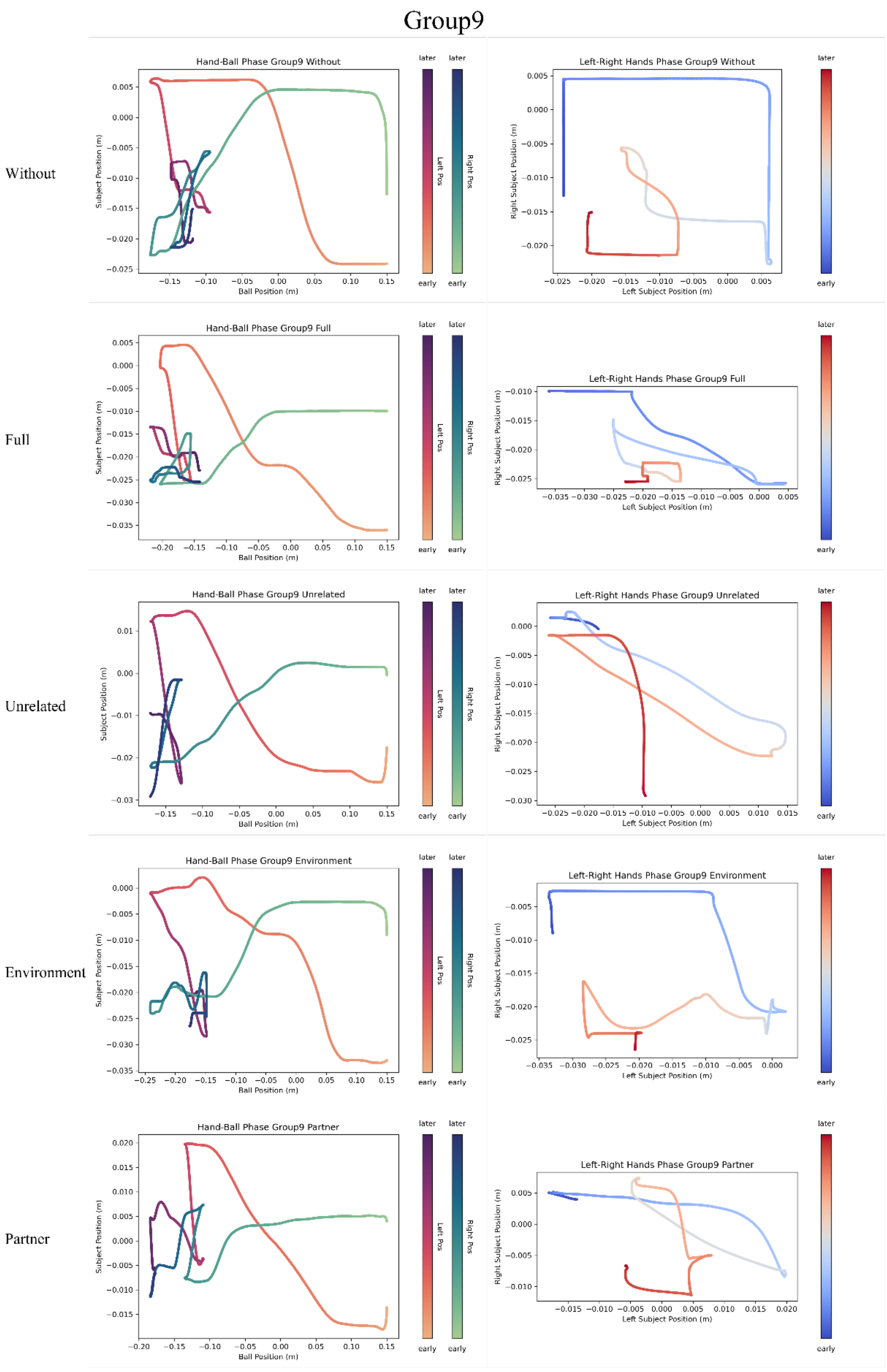

Supplementary Figure S11. Phase plots for Group 9. These plots display the relationship between hand and ball positions (Hand-Ball plots) and the relationship between the hand movements of both participants (Left-Right Hand plots). Each row represents a different haptic condition, with data taken from the final trial of each haptic condition for each dyad in the dyadic condition. Diverging colors indicate progression from early to late in the trial.

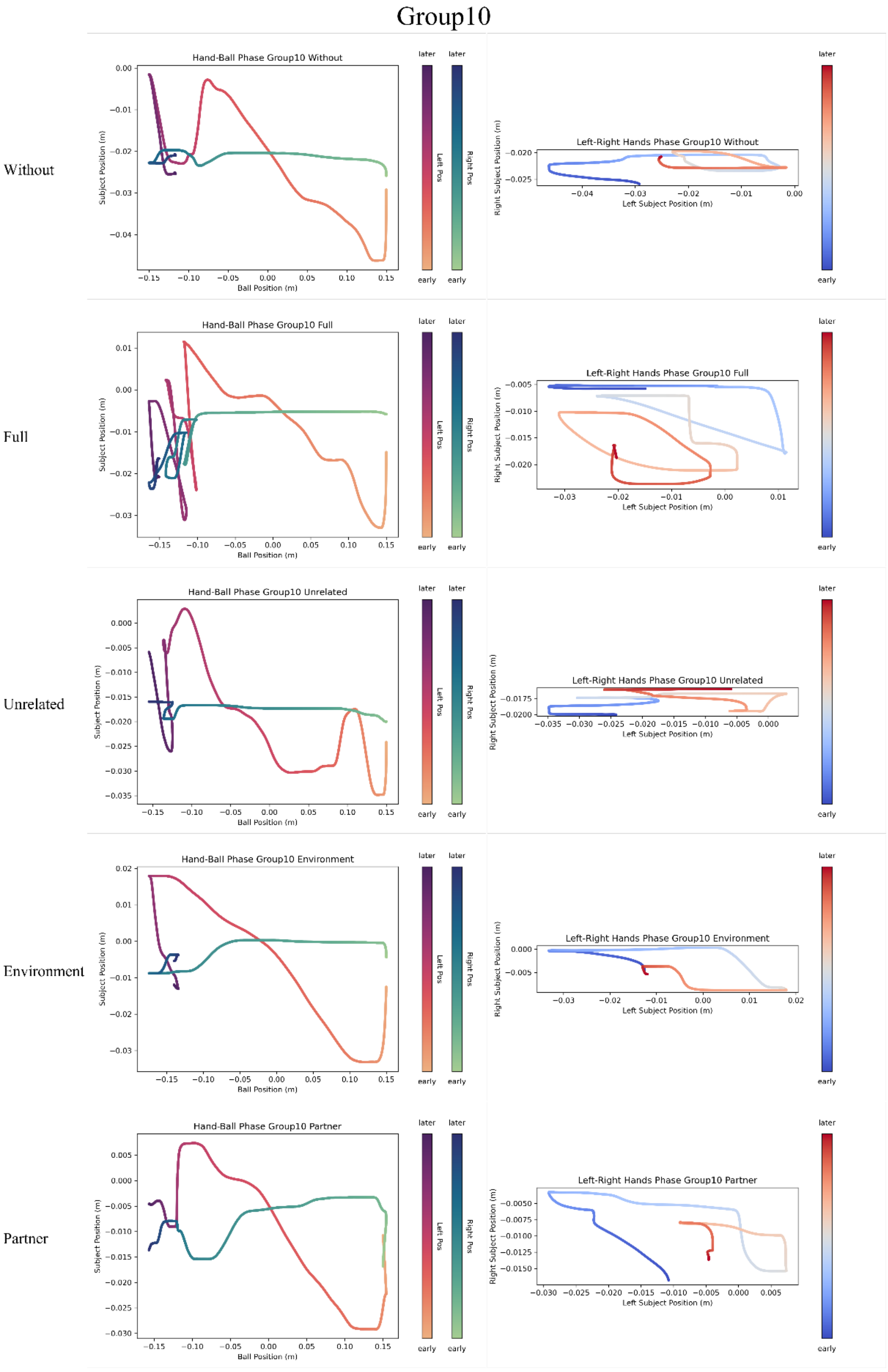

Supplementary Figure S12. Phase plots for Group 10. These plots display the relationship between hand and ball positions (Hand-Ball plots) and the relationship between the hand movements of both participants (Left-Right Hand plots). Each row represents a different haptic condition, with data taken from the final trial of each haptic condition for each dyad in the dyadic condition. Diverging colors indicate progression from early to late in the trial.
